# Inhibition of long interspersed nuclear element-1 by nucleoside reverse transcriptase inhibitors attenuates vascular calcification

**DOI:** 10.1038/s41392-025-02396-4

**Published:** 2025-10-01

**Authors:** Jianshuai Ma, Dayu He, Mingxuan Zhang, Ziting Zhou, Jinkun Cheng, Aoran Huang, Yaxin Lian, Yuncong Shi, Changming Xie, Zhengyan Guan, Zhengzhipeng Zhang, Chen Xie, Tingting Zhang, Hui Huang

**Affiliations:** Cardiology Department, the Eighth Affiliated Hospital, Joint Laboratory of Guangdong-Hong Kong-Macao Universities for Nutritional Metabolism and Precise Prevention and Control of Major Chronic Diseases, Sun Yat-sen University, Shenzhen, China

**Keywords:** Cardiology, Nephrology

## Abstract

Vascular calcification (VC) is a critical vascular pathological event, contributing to the rise in both the prevalence and fatality of cardiovascular diseases. However, the lack of effective therapeutic strategies for VC is attributed primarily to the incomplete understanding of its underlying molecular mechanisms. In this study, we discovered that long interspersed nuclear element 1 (LINE1) was significantly upregulated in the calcified arteries of both human individuals and mouse models. Mechanistically, silencing LINE1 expression or inhibiting its activity with adding nucleoside reverse transcriptase inhibitors (NRTIs, a class of validated LINE1 inhibitors) effectively prevented the osteogenic reprogramming of vascular smooth muscle cells (VSMCs). Moreover, NRTIs treatment substantially mitigated VC in chronic kidney disease (CKD)-induced and vitamin D_3_-overloaded VC mouse models. RNA sequencing analysis revealed that LINE1 depletion (via small interfering RNA) or NRTIs intervention downregulated the cGAS-STING signaling pathway and its associated inflammatory genes in VSMCs. Functional validation revealed that stimulation of the cGAS‒STING pathway exacerbated VC, whereas its pharmacological inhibition alleviated VC. Notably, we identified LINE1-derived cDNA as a direct activator of the cGAS‒STING pathway, demonstrating that LINE1 inhibition suppresses VC by blocking cGAS‒STING activation and subsequent inflammatory responses. Clinically, a cross-sectional study involving 1,785 participants revealed that patients receiving NRTIs therapy presented a significantly lower incidence of VC and reduced calcification scores. Multivariate logistic regression analysis further confirmed that NRTIs use is an independent protective factor against VC incidence and progression. Collectively, these findings establish LINE1 as promising therapeutic targets for VC and highlight NRTIs as potential candidates for developing novel strategies against VC.

## Introduction

Vascular calcification (VC) is an active process that is caused by the combined action of multiple factors and is highly regulated.^1^ VC is primarily seen in individuals with chronic kidney disease (CKD), diabetes, and atherosclerosis and serves as a unique determinant for unfavorable cardiovascular outcomes.^2^ Recent findings emphasize that VC is a pathological event defined by the alteration of vascular smooth muscle cells (VSMCs) into bone-like cells through osteogenic differentiation.^3^ Specifically, the mineralization of VSMCs is characterized by a decrease in contraction-related gene expression and an increase in osteogenic markers, both of which contribute significantly to the development of VC in the media.^4^ The osteogenic transformation of VSMCs is influenced by several pathological mechanisms, including inflammation and oxidative stress, among others.^5–7^ Recent researches have elucidated the roles of several inflammatory mediators in VC.^8^ However, the specific mechanism of VC remains unclear, and effective treatment strategies have not yet been established.

Over the past few years, transposable elements (TEs) have attracted much attention due to their important role in aging-related cellular changes.^9^ As mobile genetic elements, TEs can change their positions through DNA or RNA intermediates in the human genome.^10–12^ Although repetitive sequences derived from TEs constitute nearly 45% of the human genome, the majority have lost their ability to move due to various genetic and epigenetic alterations.^13^ Nonetheless, aging and various pathological states can activate the de-repression of TEs, which in turn leads to characteristics such as genomic instability, DNA damage, and inflammation^14^—all of which are closely associated with the onset and development of VC.^15,16^ The retrotransposon long interspersed nuclear element-1 (LINE1) represents the largest and most prevalent group of TEs capable of autonomous movement, constituting roughly 21% of human genomic DNA.^17^ Although most LINE1 elements become inactive as a result of 5’ truncation combined with the buildup of disabling, the full-length genomic LINE1 can still achieve its complete transposition through transcription by RNA polymerase II.^9^ The LINE1 messenger RNA (mRNA) is then transported to the ribosomes within the cytoplasm, where it is translated into two proteins, known as Open Reading Frames 1 and 2 (ORF1 and ORF2). The mechanism of LINE-1 transposition relies heavily on the functions of both ORF1p and ORF2p^9^ by binding to mRNAs and form LINE1 ribonucleoprotein particles (RNPs).^18^ These RNPs translocate into the nucleus during cell division and integrate into the genome through reverse transcription, potentially compromising the integrity of the host genome.^9^ Increasing evidence indicates that elevated LINE1 expression can result in the buildup of its cDNA within the cytoplasm.^19^ LINE1 activation is associated with sterile inflammation.^19–21^ Retrotransposons have been shown to exert pro-inflammatory effects in several autoimmune disorders. Increased levels of LINE1 transcripts have been identified in individuals with rheumatoid arthritis.^22^ LINE1 transcripts exhibit hypomethylation characteristics, and their transcriptional levels are significantly elevated in individuals with Sjögren’s syndrome and systemic lupus erythematosus (SLE),^23^ both of which are also characterized by the occurrence of innate immunity and inflammation. The inflammation and associated cytokines are closely intertwined with the progression of both atherosclerosis and VC.^5,24,25^ While it is known that the levels of inflammatory biomarkers in the circulatory system are related to atherosclerosis and cardiovascular disease, multiple researches have indicated that local inflammatory cytokines are key regulators in osteogenic differentiation and the progression of VC.^5^ Co-localization of inflammation and calcification was found through the detection of macrophage and osteogenic differentiation-related markers.^26^ A longitudinal study involving more than 100 patients showed clinical evidence that inflammation precedes VC—that is, the uptake of 18F-labeled glucose (a marker of metabolic activity, which may indicate inflammation) occurs in advance before radiologically visible calcification in the aortic valve annulus.^27^ Furthermore, activation of LINE1 has been associated with several diseases, such as age-related neurodegenerative conditions and cancer.^28^ However, it remains unknown whether the expression of LINE1 changes in the VC-inducing environment, whether it contributes to VC, and whether it affects the initiation and progression of VC through its role in promoting inflammatory activation.

Nucleoside reverse transcriptase inhibitors (NRTIs) are artificially analogs of nucleosides that act as competitive blockers of viral reverse transcriptases. Its strong antiviral properties have led to clinical approval for treating hepatitis B and human immunodeficiency virus (HIV), with a favorable tolerance profile observed in patients.^29^ In addition, NRTIs have been shown to inhibit macular degeneration.^30^ Notably, NRTIs suppress the reverse transcriptase activity of LINE1, which may help mitigate the effects of inflammation.^31^ However, whether NRTIs regulate the process of VC remains to be investigated.

In this research, we detected an upregulation of LINE1 in the calcified arteries of CKD patients and mice. Notably, inhibition of LINE1 by small interfering RNA (siRNA) or NRTIs alleviated the progression of VC. Mechanistically, LINE1 downregulation inhibits the cGAS-STING pathway and reduces downstream inflammatory responses, thereby alleviating VC. In a clinical cohort of 1785 patients, the use of NRTIs, which act as LINE1 inhibitors, has an independent association with the reduction in the prevalence of VC. Therefore, our study clarifies the pathogenic mechanisms of VC from the perspective of LINE1 and proposes that repurposing NRTIs, which are commonly used antiviral agents, may present a promising approach for both preventing and treating VC.

## Results

### LINE1 expression was positively related to the incidence of VC in patients with CKD

**Fig. 1:**
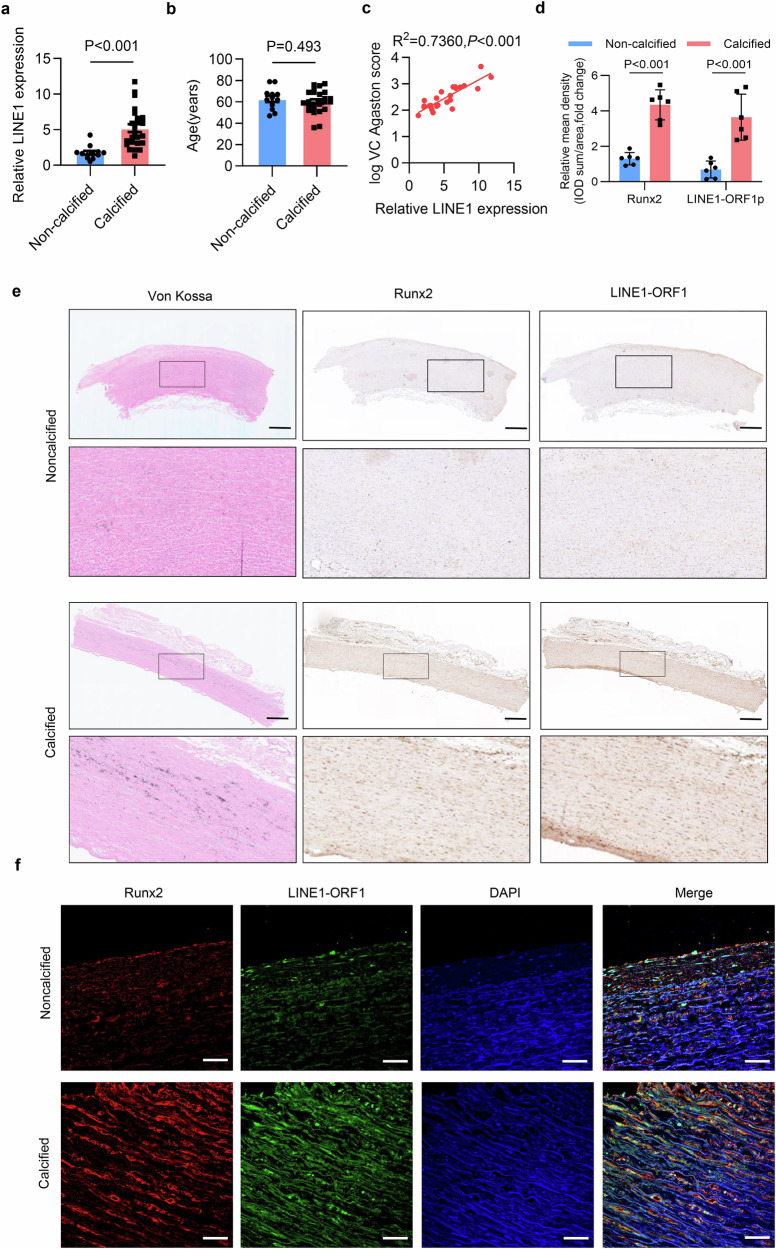
LINE1 expression was positively related to the incidence of VC in patients with CKD. **a** LINE1 mRNA levels in PBMCs of patients with CKD with (n = 25) or without(n = 12) VC. **b** Age differences between CKD patients with (n = 25) and without (n = 12) VC. **c** Correlations of LINE1 mRNA levels with VC scores (n = 25) in CKD patients (Pearson correlation coefficient *R* value and *P* value). **d**, **e** Von Kossa staining, immunohistochemical staining and quantification of Runx2 and LINE1-ORF1 in calcified and noncalcified radial artery sections (n = 6 per group), scale =100 μm. **f** Immunofluorescence staining of Runx2 and LINE1-ORF1 in calcified and noncalcified radial artery sections (n = 6 per group), scale = 50 μm. Two-tailed t-tests (**a**, **b**, **d**) and Pearson’s correlation coefficient analysis (**c**) were used to evaluate statistical significance. Data are presented as means ± SEM

To investigate the correlation between LINE1 and VC in human populations, we measured LINE1 mRNA levels in peripheral blood monocytes (PBMCs) from 37 patients with CKD. The results revealed a significant upregulation of LINE1 expression in in patients with VC (1.61 (1.13, 1.88) vs. 4.63 (3.12, 6.36), *P* < 0.001; Fig. 1a). Considering that the increase in LINE1 is related to aging, we compared the ages of patients with or without VC and found no significant difference in age (62.83 ± 10.27 vs. 60.28 ± 10.61 years, *P* = 0.493; Fig. 1b). An analysis using Spearman correlation revealed a robust positive relationship between LINE1 and the VC Agatston score (*P* < 0.001; Fig. 1c). Additionally, we investigated the relationships between LINE1 expression and the levels of Alkaline Phosphatase (ALP), Pi^+^, and Ca^2+^ in the blood of CKD patients. Our findings demonstrated a modest positive correlation between LINE1 and age (*P* = 0.0233; Supplementary Fig. 1a) as well as with the levels of ALP (*P* = 0.0226; Supplementary Fig. 1b), Pi^+^ (*P* = 0.0327; Supplementary Fig. 1d), and Ca^2+^ (*P* = 0.0406; Supplementary Fig. 1c). The baseline characteristics are detailed in Supplementary Table 1. Next, we tested LINE1 levels in human artery species by immunohistochemistry (IHC), which revealed that LINE1-ORF1 expression was prominently high in the calcified arteries (confirmed by Von Kossa staining), and was accompanied by high Runx2 levels (Fig. 1d, e). Furthermore, immunofluorescence (IF) staining demonstrated that LINE1 expression was significantly elevated and accompanied by increased Runx2 levels in the medial layer of the calcified arteries (Fig. 1f and Supplementary Fig. 1e). Overall, these data indicated that LINE1 increased in CKD patients who had VC.

### LINE1 was upregulated in calcified VSMCs and the mouse aorta

**Fig. 2:**
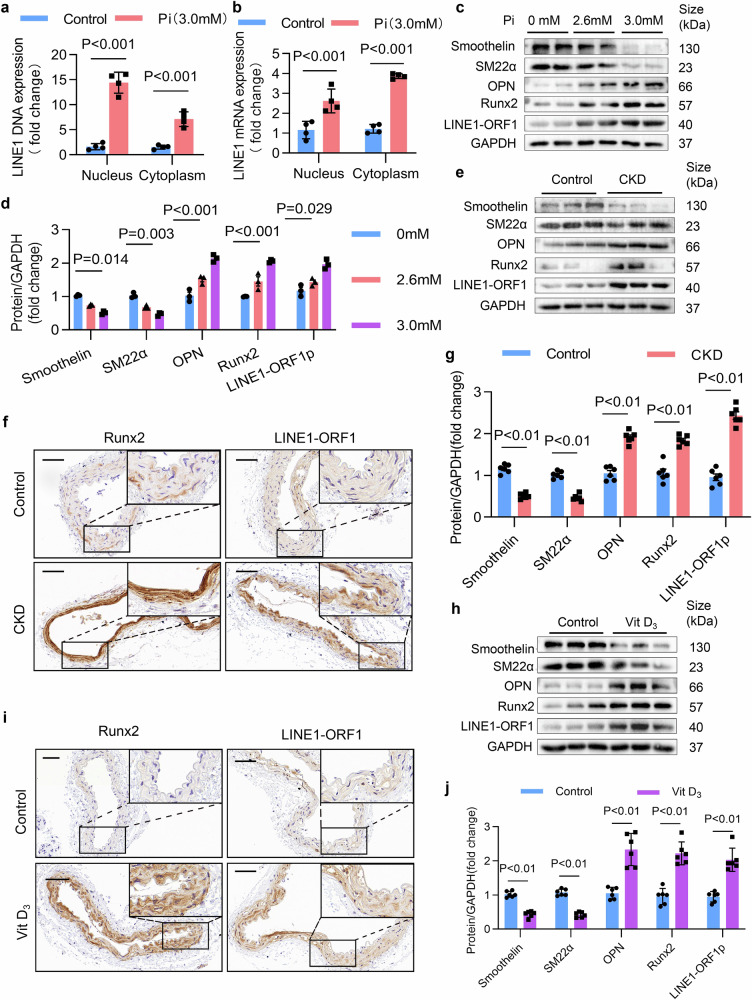
LINE1 was upregulated in calcified hVSMCs and the mouse aorta. **a**, **b** Nuclear and cytoplasmic levels of LINE1 DNA and mRNA in VSMCs exposed to Pi (n = 4 per group). **c**, **d** Western blot analysis and quantification of the expression levels of Runx2, OPN, Smoothelin, SM22α, and LINE1-ORF1 in VSMCs treated with Pi (n = 3 per group). **e**, **g** Western blot analysis and quantification of Runx2, OPN, Smoothelin, SM22α, and LINE1-ORF1 in the aortae of CKD mice (n = 6 per group). **f** Immunohistochemical staining for Runx2 and LINE1-ORF1 in aortic artery sections from CKD mice (n = 3 per group); scale bar = 100 μm. **h**, **j** Western blot analysis and quantification of Runx2, OPN, Smoothelin, SM22α, and LINE1-ORF1 expression in VitD_3_-overloaded mouse aortae (n = 6 per group). **i** Immunohistochemical staining for Runx2 and LINE1-ORF1 in aortic artery sections from VitD_3_-overloaded mice (n = 3 per group); scale = 100 μm. Statistical analysis was performed using 2-tailed t-tests (**a**, **b**, **g**, and **j**) and 1-way ANOVA followed by Dunnett’s test (**d**). All results are presented as means ± SEM

Next, we assessed LINE1 expression in vivo and in vitro. In vitro, inorganic phosphorus (Pi) successfully induced VSMCs calcification (Supplementary Fig. 2a, b). First, we quantified LINE1 DNA levels and found a notable elevation in both the cytoplasmic and nuclear fractions of calcified VSMCs compared with those of noncalcified controls (Fig. 2a). We subsequently measured LINE1 mRNA levels in both cytoplasmic and nuclear compartments, and detected a significant upregulation in calcified VSMCs (Fig. 2b). Additionally, Western blot analysis demonstrated that with the gradual increased dosage of Pi administration, LINE1-ORF1 gradually rises, along with an upregulation of osteogenic phenotypic markers (OPN and Runx2) and a decrease in contractile phenotypic markers (SM22α and Smoothelin) (Fig. 2c, d). Western blot analysis further showed a significant time-dependent increase in LINE1 expression under a time-series Pi treatment (Supplementary Fig. 2i, j). Moreover, we established two VC mouse models by a high adenine diet or vitamin D3 (VitD_3_) overload. In vivo, a high adenine diet successfully induced CKD-related VC (Supplementary Fig. 2c, d), and VitD_3_ overload induced VC (Supplementary Fig. 2g, h). Differences in renal function were found between the control and CKD, as confirmed by serum creatinine and Masson staining (Supplementary Fig. 2e, f). As anticipated, Western blot analysis demonstrated a notable increase in LINE1-ORF1 expression in the calcified aortas, accompanied by the upregulation of OPN and Runx2 but the downregulation of Smoothelin and SM22α (Fig. 2e, g). The IHC results revealed increased LINE1 and Runx2 in the arteries of calcified CKD mice (Fig. 2f). Similarly, Western blot analysis (Fig. 2h, j) and IHC (Fig. 2i) revealed elevated LINE1-ORF1 in VitD_3_-overloaded mice. In summary, these results point to the elevated LINE1 expression is present in both calcified VSMCs and mouse arteries.

### Inhibition of LINE1 alleviated VSMCs calcification

**Fig. 3:**
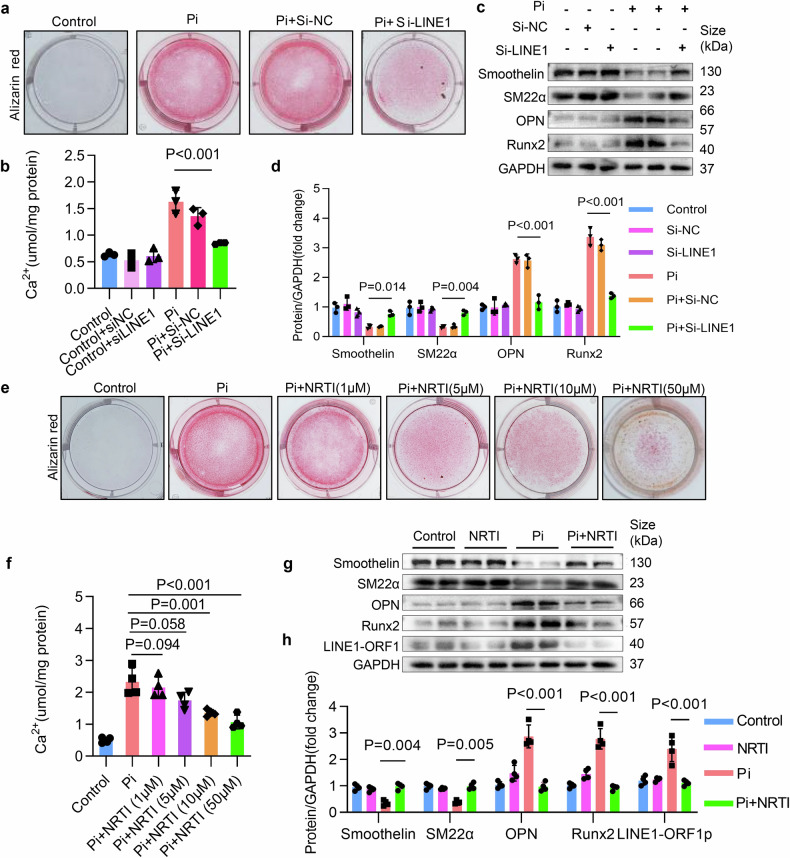
Inhibition of LINE1 alleviated VSMCs calcification. **a**–**d** Following transfection with si-LINE1, VSMCs were exposed to Pi treatment. Alizarin red staining (**a**), calcium content (**b**), Western blot (**c**), and quantitative (**d**) expression of osteogenic markers and contractile markers. VSMCs treated with Pi and NRTIs (1, 5, 10, or 50 μM). Alizarin red staining (**e**) and calcium content (**f**). Western blot analysis (**g**) and quantitative (**h**) expression of Runx2, OPN, Smoothelin, SM22α, and LINE1-ORF1 in VSMCs treated with Pi and NRTIs. Statistical significance was evaluated by 1-way ANOVA followed by Dunnett’s test (**a**, **d**, **f**, **h**). All values are displayed as the means ± SEMs

To explore the role of LINE1 in VC, VSMCs were introduced to siRNA targeting LINE1 (si-LINE1) through transfection, and the knockdown efficiency of si-LINE1 in the first and third groups were validated (Supplementary Fig. 3a–c). Alizarin red assay and calcium content measurements revealed that LINE1 knockdown effectively reduced calcium deposits (Fig. 3a, b). Moreover, Western blot analysis showed that LINE1 knockdown inhibited osteogenic markers expression while promoting contractile markers expression (Fig. 3c, d). Previous studies had confirmed that NRTIs, a group of medications employed in the management of HIV-1 and hepatitis B infections.^31^ Therefore, we investigated the role of NRTIs in VC. NRTIs notably reduced both LINE1 DNA and mRNA levels in VSMCs. (Supplementary Fig. 3d, e). As predicted, Alizarin red assay and calcium content measurements revealed that NRTIs-treated VSMCs exhibited a significant dose-dependent reduction in calcium deposition (Fig. 3e, f). Similarly, NRTIs inhibited osteogenic markers expression but promoted contractile markers expression, and LINE1-ORF1 was decreased in VSMCs (Fig. 3g, h). We also explored the role of another NRTIs drug, entecavir, in VC. Similarly, the results from calcium content measurement, alizarin red assay, and Western blot confirmed that entecavir inhibits VC (Supplementary Fig. 3f–h). Overall, we confirmed that the inhibition of LINE1 delayed VSMCs calcification.

### Inhibition of LINE1 by NRTIs alleviated VC in both CKD-induced and VitD_3_-overloaded calcified mouse models

**Fig. 4:**
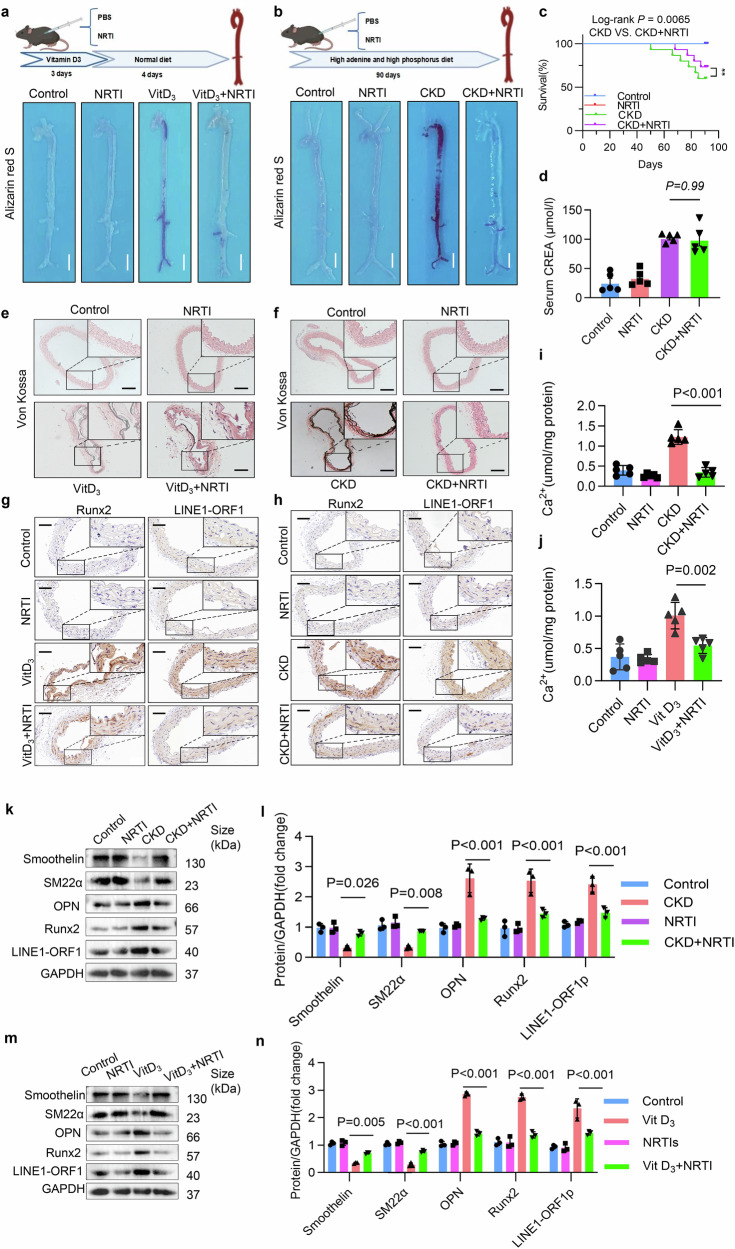
Inhibition of LINE1 by NRTIs alleviated VC in both CKD-induced and VitD_3_-overloaded calcified mouse models. Alizarin red staining of full-length aortas from CKD mice (**b**) or VitD_3_-overloaded mice (**a**) (n = 4 per group). **c** Survival curve of CKD mice treated with NRTIs. **d** Serum creatinine levels in CKD mice treated with NRTIs. Aortic cross-sections from CKD mice (**f**) or VitD_3_-overloaded mice (**e**) were stained using Von Kossa, with n = 3 per group; scale bar = 100 μm. Calcium content analysis of aortas from CKD mice (**i**) or VitD_3_-overloaded mice (**j**) (n = 5 per group). **g**, **h** Immunohistochemical staining of Runx2 and LINE1-ORF1 from CKD mice (**h**) or VitD_3_-overloaded mice (**g**) (n = 3 per group); scale = 50 μm. Osteogenic and contractile phenotypic markers, as well as LINE1-ORF1, were analyzed through Western blot (**k**) and quantitative (**l**) in CKD mice (n = 3 per group). Osteogenic and contractile phenotypic markers, along with LINE1-ORF1, were evaluated through Western blot (**m**) and quantitative (**n**) in mice with VitD_3_-overloaded (n = 3 per group). Statistical significance was evaluated by 1-way ANOVA followed by Dunnett’s test (**d**, **i**, **j**, **l**, **n**). All values are displayed as the means ± SEMs

Our aforementioned results demonstrated that NRTIs inhibit LINE1 expression in VSMCs. Consequently, we administered NRTIs to mice to evaluate the impact of LINE1 on VC. The findings indicated that NRTIs treatment notably extended the lifespan of CKD mice (Fig. 4c). However, creatinine and blood urea nitrogen (BUN) levels showed no considerable variation in renal function between the NRTI-treated and control groups (Fig. 4d and Supplementary Fig. 4a). Additionally, Masson staining and the expression of fibrosis markers revealed no notable differences in renal fibrosis between the groups (Supplementary Fig. 4b–d). In addition, alizarin red staining of the entire aorta revealed that NRTIs effectively diminished VC in both CKD and VitD_3_-overloaded mice. (Fig. 4b, a). Von Kossa staining of descending aorta sections revealed that NRTIs significantly attenuated VC in two mouse models (Fig. 4f, e). In addition, calcium content detection confirmed that NRTIs could reduce the aortic calcium content in two different mouse models of VC (Fig. 4i, j). Additionally, Western blot analysis confirmed that NRTIs inhibited LINE1-ORF1 expression in CKD mice. NRTIs treatment significantly reduced the expression of OPN and Runx2 while increasing Smoothelin and SM22α levels (Fig. 4k, l). Consistent results were observed in VitD_3_-overloaded mice via Western blot analysis (Fig. 4m, n). Furthermore, the IHC results revealed that NRTIs effectively inhibited the expression of LINE1-ORF1 and Runx2 in both CKD and VitD_3_-overloaded mice (Fig. 4h, g). In conclusion, these results suggest that NRTIs may mitigate VC by inhibiting LINE1 activation in vivo.

### Inhibition of LINE1 downregulated the cGAS-STING pathway and inflammatory genes

**Fig. 5:**
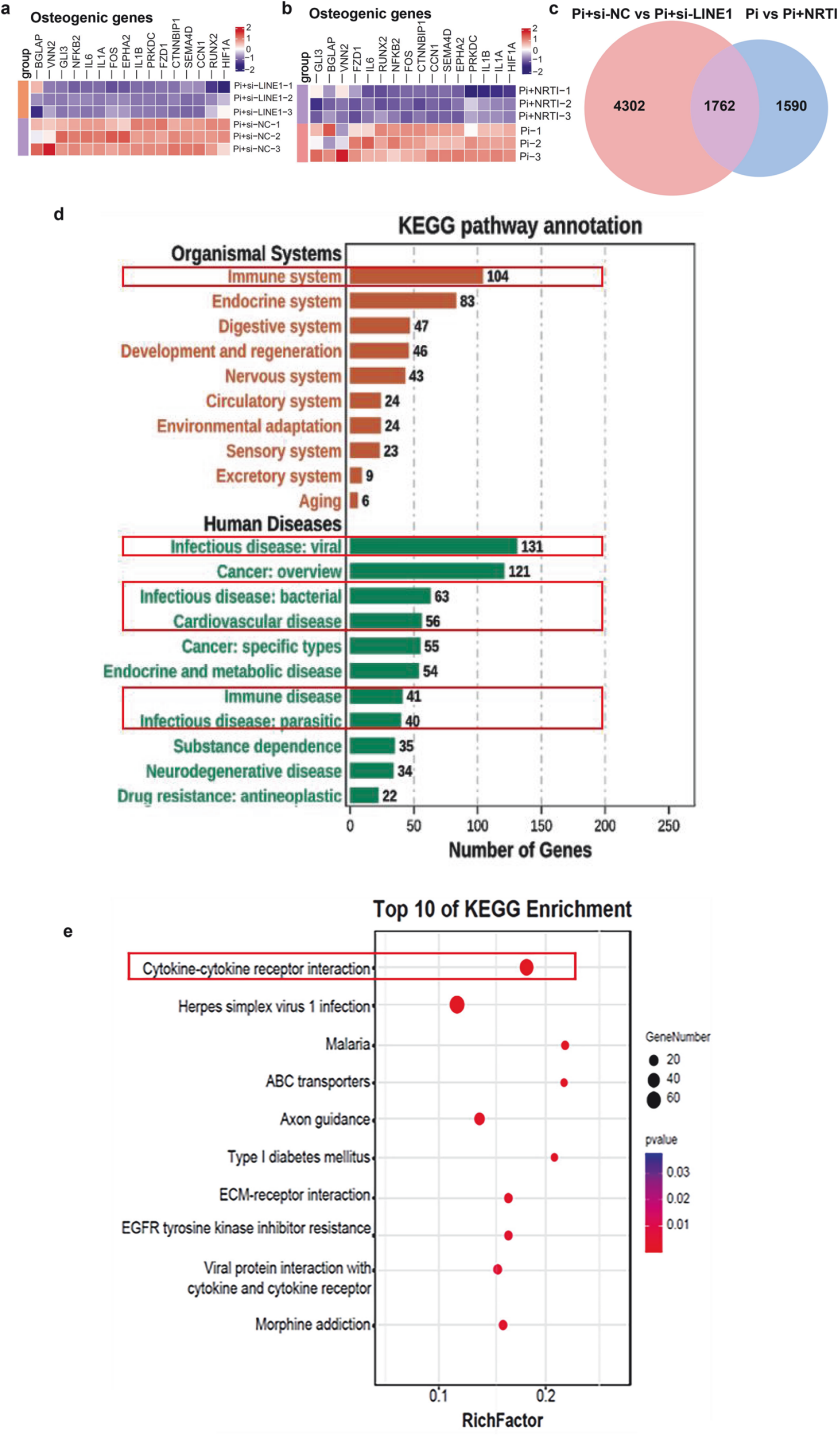

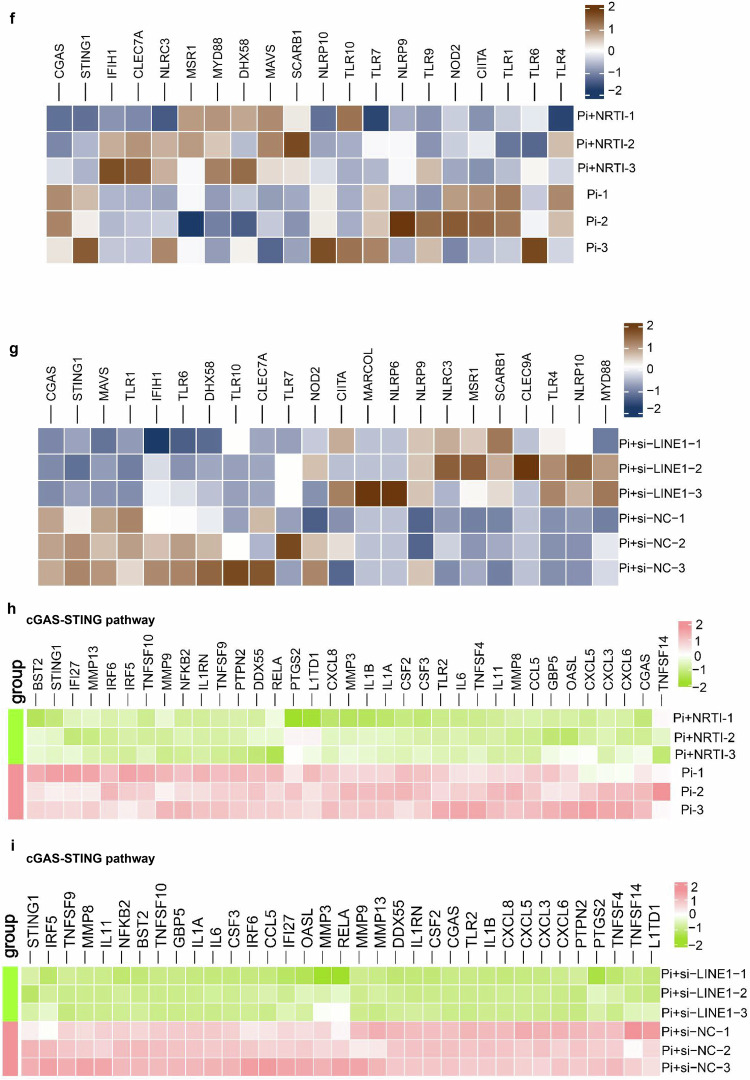
Inhibition of LINE1 downregulated the cGAS-STING pathway and inflammatory genes. Heatmap analysis of differentially expressed osteogenic genes from bulk RNA-seq of VSMCs with si-LINE1 (**a**) or with NRTIs treatment (**b**) (n = 3 per group). **c** Venn diagram showing the intersection of DEGs between two groups. **d** KEGG pathway annotation of common DEGs in both the si-LINE1 and NRTIs treatment groups resulting from bulk RNA-seq (n = 3 per group). **e** Bubble plot illustrating the top 15 most enriched KEGG pathways for common DEGs observed in both si-LINE1 and NRTIs treatment groups (n = 3 per group). Heatmap analysis of pattern recognition receptor genes from bulk RNA-seq of VSMCs with si-LINE1 (**f**) or with NRTl treatment (**g**) (n = 3 per group). Heatmap results of the cGAS-STING pathway and its downstream inflammatory factor genes from the bulk RNA-seq of VSMCs with si-LINE1(**h**) or with NRTls treatment (**i**) (n = 3 per group)

To explore the genes and associated signaling pathways involved in LINE1-regulated VC, bulk RNA sequencing was performed on Pi-induced VSMCs with LINE1 inhibition via si-LINE1 or NRTIs. Differentially expressed genes (DEGs) in VSMCs are shown in the volcano plot comparing the control with si-LINE1 (Supplementary Fig. 5a) or NRTIs groups (Supplementary Fig. 5b). Heatmaps revealed that osteogenic genes were prominently downregulated after LINE1 inhibition in both the si-LINE1 (Fig. 5a) and NRTIs (Fig. 5b) groups, whereas contractile genes were effectively increased in both the si-LINE1 (Supplementary Fig. 5c) and NRTIs (Supplementary Fig. 5d) groups. We subsequently analyzed the common DEGs that were altered by both si-LINE1 and NRTIs intervention relative to the control groups. The Venn diagram revealed 1762 common DEGs between the two groups (Fig. 5c). The gene ontology analysis indicated that common DEGs changed by si-LINE1 and NRTIs intervention were mostly enriched in the immune system and infectious disease, especially viral infection, followed by bacterial infection and immune disease (Fig. 5d). Additionally, DEGs in both the si-LINE1 and NRTIs groups were more immune- and infection-related (Supplementary Fig. 5e, f). The most prominent KEGG pathway identified from the common DEGs was the cytokine‒cytokine receptor interaction pathway (Fig. 5e). Cytokines play crucial roles in regulating immune responses, inflammation, and apoptosis through their interaction with specific receptors. Dysregulation of the cytokine‒cytokine receptor interaction pathway is closely associated with VC.^32^ These results suggest that LINE1 suppression delays calcification by inhibiting inflammation.

To further investigate the mechanisms by which LINE1 inhibition suppresses inflammation and delays calcification, we analyzed the genes affected by LINE1 suppression. Heatmaps revealed that both cGMP-AMP synthase (cGAS) and stimulator of interferon genes (STING) were significantly downregulated whether after si-LINE1 transfection or NRTIs treatment (Fig. 5f, g). The cGAS-STING is essential in mediating the inflammatory response,^33,34^ and its activation is linked to various cardiovascular disorders.^35–37^ Notably, a recent study indicated that STING knockout alleviates diabetes-associated VC in mouse models.^38^ Moreover, the heatmap demonstrated multiple genes within the cGAS‒STING pathway and downstream inflammatory factors was significantly decreased after LINE1 inhibition by si-LINE1 or NRTIs compared with that in the controls (Fig. 5h, i). These results suggest that the inflammatory response mediated by the cGAS‒STING may be a fundamental mechanism underlying the LINE1’s regulation of VC.

### Activation of the cGAS-STING pathway and excessive inflammation were required for VC

**Fig. 6:**
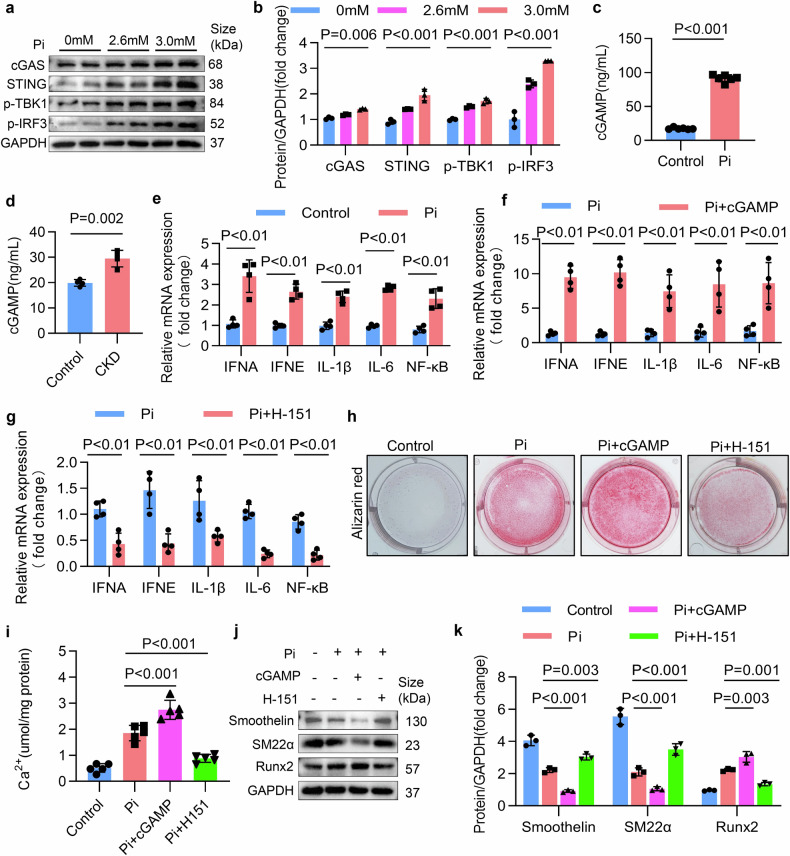
Activation of the cGAS-STING pathway and excessive inflammation were required for VC. **a**, **b** Western blot analysis and quantification of the cGAS-STING pathway-related molecules in VSMCs treated with Pi (n = 3 per group). **c**, **d** cGAMP levels in calcific VSMCs (n = 6 per group) or CKD mouse serum (n = 4 per group). **e** qPCR analysis of IFNA, IFNE, IL-1β, IL-6, and NF-κB mRNA expression in VSMCs treated with Pi (n = 4 per group). qPCR analysis of the mRNA expression of IFNA, IFNE, IL-1β, IL-6, and NF-κB in Pi-induced VSMCs treated with cGAMP (**f**) or H-151 (**g**) (n = 4 per group). Pi-induced VSMCs treated with cGAMP or H-151. **h** Alizarin red staining (n = 3 per group), **i** calcium content (n = 5 per group), **j** Western blot, and **k** quantitative (n = 3 per group) expression of osteogenic markers and contractile markers. Statistical significance was evaluated by 2-tailed t tests (**c**–**g**) and 1-way ANOVA with Dunnett’s test (**b**, **i**, **k**). All values are displayed as the means ± SEMs

As RNA-seq analysis revealed that the cGAS-STING pathway and inflammatory responses might contribute to the modulation of LINE1 on VC, we evaluated the factors related to the cGAS-STING signaling pathway in Pi-induced VSMCs. Western blot revealed that the molecules involved in the cGAS-STING were significantly increased in a manner dependent on the Pi dose (Fig. 6a, b). IF also revealed increased cGAS and STING expression in the calcified human radial artery (Supplementary Fig. 6a). Additionally, we detected 2'3’-cyclic GMP-AMP (cGAMP) levels (second messengers) in both Pi-induced VSMCs and CKD model mice and found that cGAMP levels were effectively increased in the calcified group (Fig. 6c, d). Next, we assessed several inflammatory factors closely related to the VC and cGAS-STING pathways both in Pi-induced VSMCs and in serum from CKD mice. The qPCR revealed that the mRNA expression of inflammatory molecules involved in the cGAS-STING signaling in calcified VSMCs was significantly increased (Fig. 6e), and the serum concentrations of related inflammatory mediators in CKD mice were increased (Supplementary Fig. 6b–f). Research has demonstrated that the NLRP3 inflammasome and NF-κB enhances the synthesis of pro-inflammatory cytokines thereby stimulating osteogenic activity in VSMCs through abnormal expression of RANKL and BMP2.^39–41^ In summary, our data imply that the cGAS-STING signaling cascade and inflammation are triggered in VC.

We sought to determine whether the cGAS-STING pathway contributes to the promotion of excessive inflammation, which leads to VC. We used cGAMP (a specific activator of STING)^42^ and H-151 (a specific antagonist of STING)^43^ to further interfere with the pathway in VSMCs. We tested the mRNA levels of several inflammatory molecules in calcified VSMCs and found that they were markedly increased in the cGAMP groups (Fig. 6f) but significantly decreased in the H-151 groups (Fig. 6g). These results suggest that the excessive inflammation in VC may have resulted from the stimulation of the cGAS‒STING pathway. Additionally, we explored the impact of cGAMP and H-151 on VC. Notably, alizarin red assay and calcium content analysis revealed that cGAMP accelerated calcium deposits in VSMCs, whereas H-151 alleviated calcium deposits (Fig. 6h, i). Additional Western blot results indicated that cGAMP markedly enhanced the expression of molecules associated with osteogenic phenotype (Runx2) but lowered the levels of contractile markers (Smoothelin and SM22α), whereas H-151 reversed the osteogenic change of VSMCs in Pi (Fig. 6j, k). In general, these findings imply that the stimulation of the cGAS-STING signaling axis contributes to the heightened inflammation seen in VC, ultimately promoting its progression.

### LINE1 exacerbated inflammation and contributed to VC via activation of the cGAS-STING pathway

**Fig. 7:**
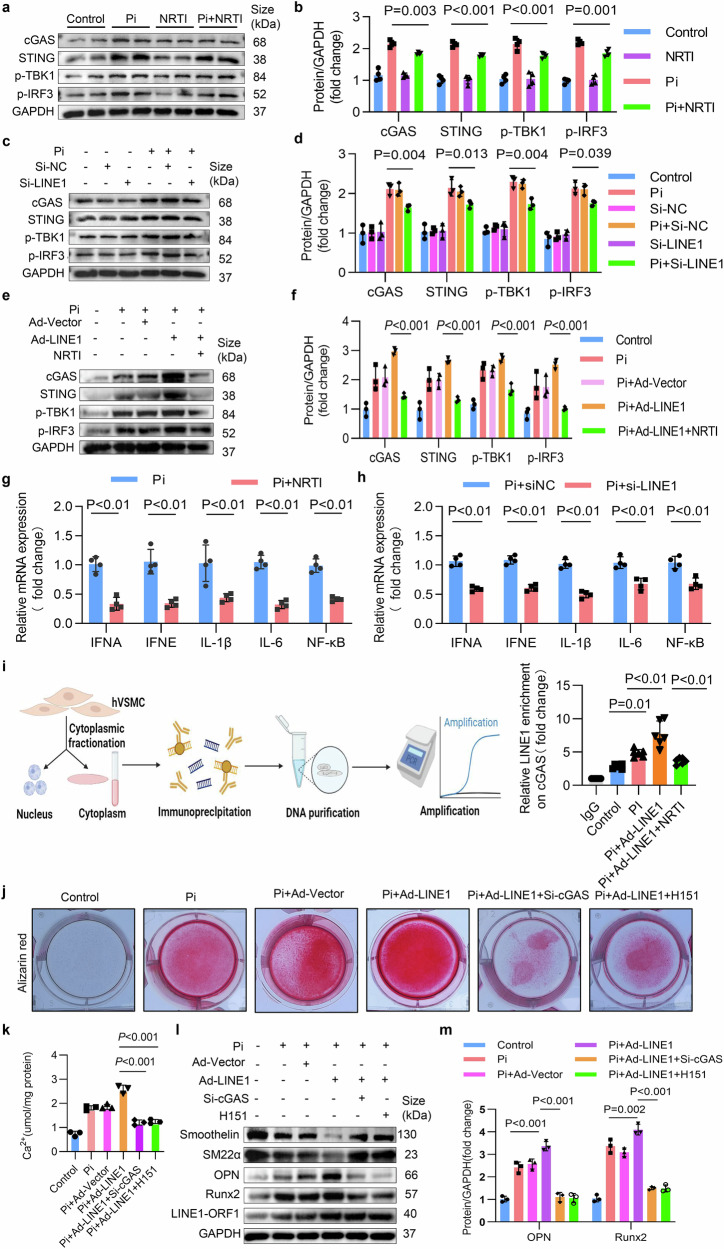
LINE1 exacerbated inflammation and contributed to VC via activation of the cGAS-STING pathway. **a**, **b** Western blot and quantitative analysis of cGAS, STING, p-TBK1, and p-IRF3 in Pi-stimulated VSMCs treated with NRTIs (n = 3 per group). **c**, **d** Western blot and quantitative analysis of cGAS, STING, p-TBK1, and p-IRF3 in Pi-stimulated VSMCs transfected with si-LINE1 (n = 3 per group). **e**, **f** Western blot and quantitative analysis of cGAS, STING, p-TBK1, and p-IRF3 in Pi-stimulated VSMCs treated with NRTIs after pretransfection with Ad-LINE1 (n = 3 per group). **g** mRNA expression of IFNA, IFNE, IL-1β, IL-6, and NF-κB in Pi induced VSMCs and treated with NRTIs (n = 4 per group). **h** mRNA expression of IFNA, IFNE, IL-1β, IL-6, and NF-κB in Pi induced VSMCs after pretransfection with si-LINE1 (n = 4 per group). **i** Chip-qPCR of cGAS and cytoplasmic LINE1 DNA in VSMCs subjected to the indicated treatments. The left panel shows a schematic diagram of the ChIP‒qPCR steps (*Created with* https://www.biorender.com/). The right panel shows the results of the ChIP‒qPCR analysis of the cytoplasmic LINE1 cDNA levels (n = 6 per group). **j**–**m** Pi induced VSMCs treated with NRTIs and pretransfected with Ad-LINE1 in the presence of si-cGAS or H-151. **j** Alizarin red staining (n = 3 per group), **k** calcium content (n = 3 per group), **l** Western blot of osteogenic phenotypic markers and contractile phenotypic markers and **m** quantitative (n = 3 per group) expression of osteogenic phenotypic markers. Statistical significance was evaluated via 2-tailed t tests (**g**, **h**) and 1-way ANOVA and Dunnett’s test (**b**, **d**, **f**, **k**, **m**). All values are displayed as the means ± SEMs

Building on these findings, we next sought to investigate whether LINE1 is the initial factor leading to excessive inflammation and whether cGAS-STING is involved in this process. Then, Western blot revealed that NRTIs inhibited the upregulation of the molecules involved in the cGAS-STING in calcified VSMCs (Fig. 7a, b, and Supplementary Fig. 7a-b). Furthermore, we silenced LINE1 expression in VSMCs using siRNA. The Western blot results revealed that levels of the molecules involved in the cGAS-STING expression in calcified VSMCs were inhibited by si-LINE1 (Fig. 7c, d, and Supplementary Fig. 7c-d). To further confirm whether LINE1-induced cGAS-STING activation contributes to inflammation in VC, we inhibited LINE1 and tested the mRNA expression of inflammatory mediators linked to the cGAS-STING signaling cascade in calcified VSMCs. The qPCR results revealed that LINE1 inhibition by NRTIs or si-LINE1 significantly decreased these mediators (Fig. 7g, h). We additionally validated the suppressive influence of NRTIs on the cGAS-STING axis and downstream inflammatory molecules in both CKD and VitD_3_-overloaded mouse aortae via Western blot (Supplementary Fig. 7g–j) and in mouse serum via ELISA (Supplementary Fig. 7k-o). We subsequently overexpressed LINE1, and the Western blot results uncovered that the cGAS-STING signaling axis (Fig. 7e, f, and Supplementary Fig. 7e, f) was significantly activated after LINE1 overexpression but prominently inhibited by NRTIs. Given these findings, we hypothesize that LINE1 overactivation may trigger the cytoplasmic DNA sensor cGAS through the buildup of its genomic components within the cytoplasm of VSMCs. As a result, this initiates the cascade of events in the entire cGAS-STING signaling axis, thereby generating proinflammatory cytokines and ultimately promoting VC. We therefore used chromatin immunoprecipitation qPCR (Chip‒qPCR) to examine the interaction between cGAS and LINE1 in the cytoplasm. The binding of cytoplasmic LINE1 cDNA to cGAS was notably higher in calcified VSMCs (Fig. 7i). This binding was further significantly enhanced after LINE1 overexpression but was notably reduced after NRTIs treatment (Fig. 7i). Furthermore, owing to LINE1 overexpression, we knocked down cGAS or inhibited the cGAS‒STING signaling axis via the inhibitor H151. As demonstrated by alizarin red assay (Fig. 7j), calcium content measurement (Fig. 7k), and Western blot assays (Fig. 7l, m), LINE1 overexpression significantly exacerbated VC. However, this effect was significantly reversed upon cGAS knockdown or H151 treatment. Overall, these findings suggest that LINE1 may serve as an initial contributor to increased inflammation in VC, which is attributed to the initiation of the cGAS‒STING.

### A clinical study revealed that NRTIs were independently associated with a lower VC incidence

**Fig. 8:**
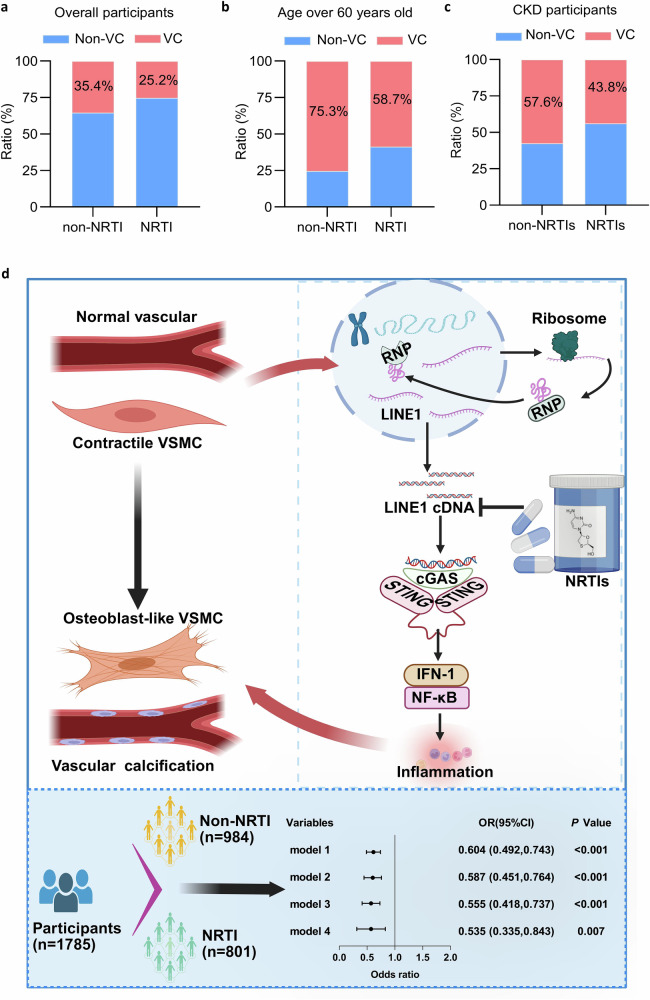
A clinical study revealed that NRTIs were independently associated with a lower VC incidence. **a**–**c** Distribution of VC between the non-NRTIs and NRTIs groups of overall participants (**a**), participants over 60 years of age (**b**), and CKD participants (**c**). **d** Graphical abstract of “*Inhibition of LINE1 by NRTIs attenuates VC by suppressing the cGAS-STING pathway”* (*Created with* https://www.biorender.com/)

A cross-sectional clinical study was conducted to further investigate the involvement of NRTIs in VC. In this study, 1785 participants were included, with males accounting for 68.5% and an average age of 51.58 years. A total of 801 patients received NRTIs treatment regularly, but 984 did not. Notably, patients taking NRTIs had a lower prevalence of VC than those did not taking (25.2% vs 35.4%, *P* < 0.001) (Table 1). Moreover, as shown in Table 1, in all stratifications of 1–100 (mild), 100–400 (moderate), and >400 (severe), the coronary artery and aortic arch calcification score in the NRTIs-taking population were lower than those in the non-medication group. Considering the established associations of VC with aging and CKD, we compared VC incidence with or without NRTIs in the general population, individuals over 60 years old, and CKD patients. Notably, NRTIs-treated patients presented a significantly lower VC prevalence across all subgroups, including the overall population, elderly individuals, and CKD patients (Fig. 8a-c).

**Table 1:** Baseline characteristics of patients with or without NRTIs use

Variables	All participants (N = 1785)	Non-NRTIs (N = 984)	NRTIs (N = 801)	*P* Value
Age, years	51.58 ± 13.22	52.32 ± 13.41	50.67 ± 12.97	0.009
Male, n (%)	1224 (68.5%)	636 (64.2%)	588 (73.8%)	<0.001
Diabetes, n (%)	129 (7.2%)	57 (5.8%)	72 (9.0%)	0.009
CKD, n (%)	221 (12.4%)	125 (12.6%)	96 (12.1%)	0.754
CVD, n (%)	357 (20.0%)	221 (21.4%)	146 (18.2%)	0.091
Hypertension, n (%)	75 (4.2%)	41 (4.2%)	34 (4.2%)	0.935
Total cholesterol, mmol/L	4.62 (3.89,5.47)	4.62 (3.88, 5.42)	4.61 (3.90, 5.49)	0.522
Triglycerides, mmol/L	1.27 (0.94,1.76)	1.25 (0.92, 1.74)	1.29 (0.96, 1.80)	0.106
LDL-C,mmol/L	2.85 (2.30,3.46)	2.82 (2.28, 3.41)	2.88 (2.32, 3.51)	0.129
HDL-C,mmol/L	1.12 (0.92,1.34)	1.12 (0.92, 1.35)	1.11 (0.91, 1.32)	0.517
APOa, mg/dl	123.32 ± 30.42	123.88 ± 29.23	122.64 ± 31.84	0.432
APOb, mg/dl	85.46 (67.49,102.28)	83.94 (65.36, 100.46)	87.18 (69.62, 104.83)	0.001
CREA,μmol/L	74.99 (61.04,90.61)	74.85 (59.86, 89.92)	75.25 (63.00, 92.90)	0.393
HGB(g/L)	137.17 ± 20.01	136.54 ± 19.85	137.94 ± 20.23	0.149
PLT(X10^9/L)	221.76 ± 84.01	229.07 ± 81.87	211.71 ± 86.03	0.002
TP(g/L)	69.71 (65.39,74.14)	69.59 (65.34, 73.90)	69.90 (65.53, 74.50)	0.326
ALP(U/L)	40.75 (37.62,43.87)	40.86 (37.92, 43.90)	40.48 (37.22, 43.80)	0.119
TBIL(μmol/L)	14.70 (10.79,21.15)	14.10 (10.48, 19.39)	15.54 (11.30, 24.60)	<0.001
DBILL(μmol/L)	2.85 (2.03,4.50)	2.69 (1.97, 3.94)	3.21 (2.12, 5.50)	<0.001
ALT(U/L)	29.70 (18.66,59.70)	27.10 (17.41, 48.00)	34.27 (20.32, 83.30)	<0.001
AST(U/L)	28.64 (21.00,53.79)	26.30 (20.72, 41.96)	33.27 (21.87, 73.56)	<0.001
VC, n (%)	552 (30.9%)	351 (35.4%)	201 (25.2%)	<0.001
BMI, (kg/m^2^)	22.58 ± 10.32	22.95 ± 11.09	22.14 ± 9.28	0.094
eGFR (mL/min/1.73 m²)	89.18 ± 32.61	88.01 ± 32.55	90.60 ± 32.65	0.100
**Aortic Artery Calcium Score**				
0–100	60.71 (20.71,78.71)	72.58 (34.58,82.50)	43.94 (14.86,71.27)	0.013
101–400	251.43 (173.57,342.00)	282.66 (202.50,347.27)	219.75 (143.21,281.78)	0.010
>400	1383.57 (761.33,2616.56)	1512.14 (829.69,2738.57)	1190.32 (678.93,2172.86)	0.026
**Coronary Artery Calcium Score**				
0–100	21.21 (9.39,56.89)	24.24 (11.53,67.41)	15.9 (7.00,40.25)	0.010
101–400	248.26 (177.00,318.70)	259.89 (214.50,325.94)	218.25 (153.47,303.08)	0.023
>400	1152.17 (644.74,2479.55)	1332.47 (752.15,2522.12)	796.49 (557.10,2160.53)	0.030

Categorical variables are presented as n (%), while continuous variables are expressed as either mean ± SD or median (25th, 75th percentiles)

*NRTIs* nucleoside reverse transcriptase inhibitors, *CKD* chronic kidney disease, *CVD* cardiovascular disease, *LDL-C* low-density lipoprotein cholesterol, *HDL-C* high-density lipoprotein cholesterol, *APOa* Apolipoprotein A, *APOb* Apolipoprotein B, *CREA* creatinine; *HGB* hemoglobin; *PLT* platelets, *TP* total protein, *ALP* alkaline phosphatase, *TBIL* total bilirubin, *DBIL* direct bilirubin, *ALT* alanine transaminase, *AST* aspartate transaminase, *VC* vascular calcification

To elucidate the association between NRTIs and VC, we conducted logistic regression analyses both univariate and multivariate. Table 2 demonstrates that the use of NRTIs was independently linked to a reduced prevalence of VC in several adjusted models. In Model 1, the unadjusted odds ratio (OR) was 0.604 (95% CI: 0.492–0.743, P < 0.001). Model 2 revealed an odds ratio (OR) of 0.587 (95% CI: 0.451–0.764, P < 0.001) after adjusting for sex, age, diabetes status, CKD, CVD, hypertension, eGFR, and body mass index. Model 3, which further incorporated lipid parameters (triglycerides, low-density and high-density lipoproteins, apolipoproteins, and total cholesterol) and creatinine, yielded an OR of 0.555 (95% CI: 0.418–0.737, P < 0.001). In the final analysis, adjustments for all variables in Model 3, along with liver function markers (total and direct bilirubin, AST, ALT) and hematological parameters (hemoglobin and platelets), were incorporated into Model 4, resulting in an OR of 0.535 (95% CI: 0.335–0.843, P = 0.007) (Table 2).

**Table 2:** Univariate and multivariate logistic regression analyses on the association between NRTIs treatment and VC incidence

Variables	OR	95% CI	*P* Value
**Model1**	0.604	0.492–0.743	<0.001
**Model2**	0.587	0.451–0.764	<0.001
**Model3**	0.555	0.418–0.737	<0.001
**Model4**	0.535	0.335–0.843	0.007

**Model 1** unadjusted

**Model 2** was adjusted for age, sex, cardiovascular disease(CVD), chronic kidney disease(CKD), diabetes, hypertension, body mass index(BMI), and estimated glomerular filtration rate (eGFR)

**Model 3** was adjusted for the variables in Model 2 and total cholesterol, triglyceride, low-density lipoprotein cholesterol (LDL-C), high-density lipoprotein cholesterol (HDL-C), apolipoprotein A, apolipoprotein B, and creatinine

**Model 4** was adjusted for variables in Model 3 and included hemoglobin, platelets, total protein, alkaline phosphatase, total bilirubin, direct bilirubin, alanine transaminase (ALT), and aspartate transaminase (AST)

*CI* confidence interval, *OR* odds ratio, *NRTIs* nucleoside reverse transcriptase inhibitor, *VC* vascular calcification

Multivariate logistic regression analysis was employed to explore the association between LINE1 inhibitor NRTIs and the severity of VC, with the calcification score serving as the basis for assessment. The results in Table 3 revealed that, compared with the non-medication group, the risk of both aortic and coronary artery calcification was significantly lower in patients taking NRTIs, and this risk reduction was observed across different severity levels of calcification, including stratifications of 1–100 (mild), 101–400 (moderate), and >400 (severe). These findings indicate that NRTIs use is an independent protective factor for VC prevention.

**Table 3:** Multivariate logistic regression analyses on NRTIs treatment and the severity of VC

Subgroups	OR	95% CI	*P* Value
**Aortic Artery Calcium Score**			
1–100	0.578	0.377–0.887	0.012
101–400	0.544	0.352–0.841	0.006
>400	0.502	0.341–0.738	<0.001
1–100 VS > 400	1.153	0.693–1.919	0.584
101–400 VS 1–100	0.909	0.514–1.607	0.742
101–400 VS > 400	1.085	0.668–1.761	0.742
**Coronary Artery Calcium Score**			
1–100	0.613	0.414–0.909	0.015
101–400	0.552	0.357–0.852	0.007
>400	0.499	0.326–0.765	0.001
1–100 VS > 400	1.228	0.737–2.045	0.430
101–400 VS 1–100	1.069	0.623–1.835	0.809
101–400 VS > 400	1.105	0.673–1.816	0.692

All the results were adjusted for potential confounders including age, sex, cardiovascular disease, chronic kidney disease, diabetes, hypertension, body mass index (BMI), estimated glomerular filtration rate (eGFR), triglycerides, total cholesterol, low-density lipoprotein cholesterol (LDL-C), high-density lipoprotein cholesterol (HDL-C), apolipoprotein A, apolipoprotein B, creatinine, alanine transaminase (ALT), and aspartate transaminase (AST)

*CI* confidence interval, *OR* odds ratio, *NRTIs* nucleoside reverse transcriptase inhibitors, *VC* vascular calcification

To identify populations that may benefit from NRTIs treatment, we subsequently performed multivariate logistic regression to analyze the risk of VC in distinct subgroups of NRTIs users versus nonusers, adjusting for traditional VC risk factors. As shown in Table 4, in both patients aged over 60 years (OR 0.410; 95% CI: 0.256–0.685; *P* = < 0.001) and those aged younger than 60 years (OR 0.654; 95% CI: 0.455–0.939; *P* = 0.021), the VC risk was notably lower in NRTIs users than in nonusers. In both populations with CKD (OR 0.383; 95% CI: 0.181–0.812; *P* = 0.012) and without CKD (OR 0.628; 95% CI: 0.476–0.830; *P* = 0.001), individuals using NRTIs exhibited a substantially reduced risk of VC than nonusers did. In individuals without diabetes (OR 0.555; 95% CI: 0.407–0.756; *P* < 0.001) or without CVD (OR 0.500; 95% CI: 0.349–0.715; *P* < 0.001), the risk of VC was notably reduced in individuals using NRTIs compared to those who did not use them. However, in populations with diabetes or CVD, no difference in calcification risk was found between NRTIs users and non-users.

**Table 4:** Subgroup analyses on the association between NRTIs treatment and the incidence of VC

Subgroups	OR	95% CI	*P* Value
**Age, years (%)**			
≥60 (26.9)	0.410	0.256–0.685	<0.001
<60 (73.1)	0.654	0.455–0.939	0.021
**SEX (%)**			
Male (68.5)	0.526	0.372–0.744	<0.001
Female (31.5)	0.573	0.315–1.041	0.067
**CKD (%)**			
YES (12.4)	0.383	0.181–0.812	0.012
NO (87.6)	0.628	0.476–0.830	0.001
**Diabetes (%)**			
YES (7.2)	0.340	0.082–1.413	0.138
NO (92.8)	0.555	0.407–0.756	< 0.001
**CVD (%)**			
YES (20.0)	0.577	0.330–1.009	0.054
NO (80.0)	0.500	0.349–0.715	< 0.001

All the results were adjusted for factors such as age, sex, cardiovascular disease (CVD), chronic kidney disease (CKD), diabetes, hypertension, body mass index (BMI), estimated glomerular filtration rate (eGFR), triglycerides, total cholesterol, low-density lipoprotein cholesterol (LDL-C), high-density lipoprotein cholesterol (HDL-C), alkaline phosphatase, total bilirubin, direct bilirubin, alanine transaminase (ALT), and aspartate transaminase (AST)

*CI* confidence interval, *OR* odds ratio, *NRTIs* nucleoside reverse transcriptase inhibitors, *VC* vascular calcification

Above all, our findings demonstrate that NRTIs treatment is linked to a lower incidence and severity of VC. These findings imply that NRTIs could be promising therapeutic options for VC prevention and management

## Discussion

In this research, we elucidated the pivotal involvement of LINE1 in VC. Our findings revealed significant upregulation of LINE1 levels in calcified arteries from CKD patients and mice. Notably, LINE1 inhibition alleviated VC in vitro and in vivo. Mechanistically, bulk RNA sequence analysis indicated that LINE1 inhibition downregulated the cGAS‒STING signaling cascade and the downstream inflammatory genes it regulates in calcified VSMCs. Further intervention experiments confirmed that the initiation of the cGAS-STING signaling pathway and excessive inflammation are critical for the progression of VC. In addition, we recognized that cytoplasmic LINE1 cDNA in calcified VSMCs is a vital component triggering the cGAS-STING pathway. In our clinical research, we further confirmed that the use of NRTIs is independently linked to a lower prevalence of VC, highlighting their potential as promising candidates for treatment.

Previous studies have indicated that LINE1 expression increases in aging cells and organisms, suggesting its potential role as a driver of age-related diseases.^44,45^ However, the relationship between LINE1 and VC, which is a manifestation of vascular aging in CKD patients,^46^ remains unclear. Research has shown elevated LINE1 levels within the circulatory system of individuals experiencing stroke and ischemic heart disease.^47,48^ Our study is consistent with these findings, demonstrating that LINE1 is upregulated in VC within humans and mice and that LINE1 is positively correlated with the severity of calcification. Our findings are reinforced by a cohort study involving 794 individuals diagnosed with type 2 diabetes, which revealed that elevated LINE1 methylation was correlated with decreased diastolic blood pressure, eGFR, and HDL cholesterol ratios.^49^ These associations underscore a potential link between LINE1 and VC and highlight the novel role of LINE1 in VC. Recently, the modulation of LINE1 activation as a means to mitigate aging has garnered increasing attention in the life sciences.^50^ Previous studies have demonstrated that inhibiting LINE1 reverse transcriptase activity via NRTIs or silencing LINE1 expression via short hairpin RNA or siRNA effectively reduces cellular LINE1 levels and alleviates aging-related phenotypes.^31,51^ Moreover, LINE1-specific antisense oligonucleotide therapy has been shown to lower LINE1 RNA levels, thereby improving the expression of aging markers.^45^ Notably, VC often worsens with aging.^52^ However, whether LINE1 suppression can directly alleviate VC remains to be elucidated. Our findings indicate that knocking down LINE1 via siRNA or administering NRTIs significantly diminishes osteogenic transdifferentiation in VSMCs. Additionally, in CKD and VitD_3_-overloaded mouse models, NRTIs treatment effectively alleviated VC. Elevated calcium and phosphate are reported to cause the loss of heterochromatin, which leads to VC.^53^ Silencing LINE1 is associated with heterochromatinization at these sites, indicating that increased phosphate may affect LINE1 through modulating the loss of heterochromatin.^54^ Moreover, the process of heterochromatinization involves the activation of chromatin-remodeling enzymes and the recruitment of effector proteins.^9^ As one of these key proteins involved in maintaining heterochromatin, SIRT6 can potently inhibit LINE1 expression.^55^ In addition, SIRT6 is inhibited under conditions of high phosphate levels or other calcification-related factors, and loss of SIRT6 exacerbates VC.^56^ Therefore, high Pi may activate LINE1 by inhibiting the SIRT6-mediated loss of heterochromatin, which in turn promotes VSMCs calcification. This may be one of the mechanisms by which Pi activates LINE1. Together, these results emphasize the key role of LINE1 in VC progression and support the therapeutic potential of LINE1 intervention in mitigating VC.

The dysregulation of LINE1 has been linked to the stimulation of innate immune responses, which can lead to DNA damage and cell death, ultimately promoting cellular senescence.^9^ However, the precise mechanisms by which LINE1 inhibition alleviates VC remain unclear, and our transcriptomic analysis provides critical insights. Our bulk RNA-seq results from calcified VSMCs with LINE1 inhibition by siRNA or NRTIs revealed a significant downregulation of inflammation-related responses. This observation aligns with the established understanding that inflammation is a hallmark of VC,^57–59^ although the specific molecular pathways mediating the aberrant inflammatory state in VC are yet to be fully elucidated. Our results underscore that cGAS-STING activation and subsequent inflammatory cascades are required for VC. The hyperactivation of cGAS-STING induced by LINE1 dysregulation plays a crucial role in the senescence process.^60,61^ Moreover, we revealed a notable increase in LINE1 cDNA binding to cGAS in the cytoplasm of calcified VSMCs, as confirmed by ChIP‒qPCR, which was mitigated by NRTIs treatment. Importantly, both siRNA and NRTIs significantly reversed the initiation of the cGAS-STING signaling cascade, mitigating inflammation in VC. In conclusion, our research demonstrated the cGAS-STING-mediated regulation in the inflammatory environment of calcified VSMCs and highlighted LINE1-derived DNA as a significant contributor to inflammation and VC.

NRTIs have long been recognized for their efficacy in treating hepatitis B and HIV.^62^ Recent studies, however, have prompted a reevaluation of their potential beyond antiviral therapy, particularly in relation to aging and associated diseases.^31^ Notably, NRTIs has the capacity to mitigate aging processes in various cellular and animal models, as evidenced by findings in BMAL1 knockout human mesenchymal stem cells and SIRT6 knockout mice.^20,63^ Clinically, data indicate that patients receiving NRTI treatment exhibit a markedly reduced risk of developing hypertension (25.89% vs. 15.1%), hypertriglyceridemia (10.01% vs. 6.59%), hypercholesterolemia (2.17% vs. 1.26%), and ischemic heart disease (4.15% vs. 2.12%) than those not on these medications.^64^ Importantly, our study involving 1,785 individuals revealed a novel association between NRTIs use and reduced VC. We identified NRTIs as an independent protective factor against VC. However, in populations with diabetes, calcification risk did not differ significantly between NRTIs users and nonusers. Indeed, the mechanisms of VC differ between diabetic patients and CKD patients. In diabetic patients, under conditions of hyperglycemia and insulin resistance, calcification primarily involves the advanced glycation end products (AGEs) deposition and endothelial dysfunction. These effectors induce VSMCs switching to osteoblast-like cells and accelerate calcium salt deposition.^65–68^ In CKD patients, VC is more closely linked to decreased renal function, increased phosphorus load, abnormal vitamin D metabolism, and disturbances in calcium‒phosphate homeostasis.^1,2^ Thus, the divergent mechanisms of VC between diabetic and CKD patients may explain why we did not observe a protective effect of NRTIs in the diabetic subgroup. Additionally, prior epidemiological studies highlighted that in a cohort of 128,861 patients with HIV-1 or hepatitis B, a 33% lower adjusted risk of diabetes was linked to NRTIs use.^69^ Furthermore, an analysis of 72,193 HIV or hepatitis B patients without a history of Alzheimer’s disease indicated that NRTIs exposure demonstrated an association with decreased Alzheimer’s disease risk, alongside improved survival probabilities, as evidenced by Kaplan‒Meier survival analysis.^70^ Collectively, these findings suggest that NRTIs, such as VC, have significant potential for repurposing in the clinical setting. However, this cross-sectional analysis has inherent limitations and cannot directly establish a causal relationship between LINE1 and VC. Additionally, since patients in this study who took the same drug almost all received substantially identical dosages, this clinical study was unable to conduct dose-dependent related research. Longitudinal prospective cohort clinical trials are needed to comprehensively evaluate factors such as treatment duration, drug types and dosages, efficacy, and safety of NRTIs in VC therapy and investigating the potential causal link between LINE1 and VC.

In conclusion, we discovered a new function of LINE1 in VC, particularly through its effect in activating the cGAS-STING pathway, which results in the upregulation of downstream inflammatory molecules and thus leads to VC. Importantly, our findings indicate that NRTIs inhibit LINE1 activation, thereby alleviating VC (Fig. 8d). This study not only deepens our comprehension of the mechanisms involved in VC but also indicates that NRTIs may offer a therapeutic approach for both preventing and managing this condition. Future studies should explore the clinical applicability of NRTIs in managing VC and related vascular disorders.

## Materials and methods

### Ethical approval

The human primary VSMCs used in the cell experiment were sourced from the American Type Culture Collection (ATCC). Animal studies received ethical authorization (TOP-IACUC-2023-0324) from Shenzhen TopBiotech Co., Ltd., conducted in accordance with National Institutes of Health for the Care and Use of Laboratory Animals (8th Edition, 2011). Human specimen research adhered to Helsinki Declaration principles under ethical clearance granted by Donghua Hospital Institutional Review Board, Sun Yat-sen University (SYSEC-KY-KS-2020-191). The informed consent of all participants who were included in the study had been obtained prior to the research. The clinical data were obtained from the Eighth Affiliated Hospital of Sun Yat-sen University and were sanctioned by the Institutional Review Board of the Eighth Affiliated Hospital of Sun Yat-sen University [EZ-KYIRB-AF/SC-08/02.0].

### Cell culture

Gibco’s high-glucose Dulbecco’s modified Eagle’s medium (DMEM) was used to culture the hVSMCs and added with 10% fetal bovine serum (FBS, MIKX) as well as 1% penicillin/streptomycin (P/S, Gibco). Experiments exclusively utilized passages 5–10 cells. To promote the formation of VSMCs calcification, subconfluent VSMCs (60% confluence) were exposed to 3.0 mmol/L inorganic phosphate (Pi) in complete medium, with medium replenishment performed every 2 days. The Pi solution was composed of sodium phosphate dibasic solution (0.5 mol/L in H_2_O; BioUltra) purchased from SigmaAldrich (94046-100ML-F). In accordance with the manufacturer’s guidelines (Invitrogen, USA, catalog number 13778150), VSMCs were transfected with siRNA. We use Lipofectamine RNAIMAXQ6 to transfect the LINE1 siRNAs (50 nM, IGE Biotechnology, China). siRNA sequences are detailed in Supplementary Table 2. Treatments were carried out simultaneously with Pi stimulation: NRTIs (Lamivudine, MedChemExpress, USA, HY-B0250), cGAMP (MedChemExpress, USA, HY-12512), and H-151 (MedChemExpress, USA, HY-112693) were selected as the working concentrations.

### Animal experiment

Male C57BL/6J mice, aged 8 weeks and weighing 20–25 g, were obtained from the Laboratory Animal Center at Sun Yat-Sen University. Housing conditions comprised a controlled environment set to 23–25 °C with humidity control, under a 12 h light/dark photoperiod. Random assignment was made of the mice into various experimental groups, each containing 15 mice. To model CKD-associated VC, the mice were fed a diet rich in adenine (0.15%) and phosphorus (1.9%) for 12 weeks (AP diet). In an alternative approach, VC was induced by administering intraperitoneal injections of VitD_3_ (500 IU/kg) daily for three consecutive days. Following the injections, the mice were switched to a standard chow diet until they were euthanized on the 10th day for further analysis. Simultaneously, the mice were administered daily intraperitoneal injections of either NRTIs (1 mg/kg) or a vehicle control (1 ml/kg). Terminal procedures initiated with isoflurane anesthesia (5% induction, 2% maintenance), followed by peripheral blood collection for serum creatinine quantification and ELISA profiling. After a lethal dose of isoflurane was reached, the aorta and kidneys were excised for subsequent analysis.

### Human samples and clinical data

Between November 2019 and January 2020, peripheral blood was collected from 37 CKD subjects at Donghua Hospital of Sun Yat-sen University. Patients underwent chest multidetector computed tomography scanning to assess thoracic aorta calcification. Three independent investigators, blinded to patient information, used Siemens Syngo CT Workplace software to calculate Agatston scores on the basis of established criteria. The area between the ascending aorta and the descending aorta is regarded as the thoracic aorta. To assess the degree of calcification, we reconstructed the CT images with a layer thickness of 1 mm. In this study, the degree of calcification was determined by the Agatston score. Peripheral blood mononuclear cells (PBMCs) were separated for RNA analysis. The blood sample was processed by centrifugation at 400 × *g* for 20 min, and the PBMCs were retrieved from the interface layer. We obtained the clinical and biochemical data from the patient’s Inpatient Medical Record System. Additionally, radial artery samples from patients with CKD were collected during the same period at the Donghua Hospital of Sun Yat-sen University. From patients with CKD who had undergone arteriovenous fistula surgery (among them, six patients were diagnosed with aortic arch calcification, the calcification group;6 additional upper limb trauma amputation patients (absence of CKD/diabetes diagnosis) comprised the non-calcification group), 4–6 mm long radial arteries were extracted.

Data for the clinical study were collected from patients with AIDS and viral hepatitis B who were hospitalized at the Eighth Affiliated Hospital of Sun Yat-sen University between September 2018 and October 2023. After patients who had not received CT or X-ray data were excluded, we included a number of 1785 patients were included. According to the inpatient doctor’s advice system, the patients were divided into 801 patients who took NRTIs and 984 patients who did not take NRTIs. In this research group, all patients underwent MDCT scans of the chest. We used a CT attenuation threshold of >130 HU, a criterion widely applied in coronary artery calcification scoring. The Agaston scores for all images were quantified using the Siemens Syngo CT Workplace software in accordance with the standard criteria.^71^ Anatomically, the aortic arch constitutes the segment bridging the ascending and descending aorta. Calcification burden was quantified through 1-mm slice thickness CT reconstructions. Per study criteria, vascular calcification positivity required an Agatston score >0. Both aortic arch and coronary artery calcifications were stratified into three severity tiers: mild, moderate, and severe.^72–74^ Patient demographic and clinical variables were retrieved from institutional electronic medical records. To address missing data, we employed multiple imputation, a commonly used technique in clinical research that helps minimize bias associated with absent data. The data after imputation were then utilized for all subsequent statistical analyses.

### Western blot

Following the addition of ice-cold RIPA buffer to harvested cells and arterial samples, tissues were homogenized using cryogenic grinding. Lysates were aliquoted into 1.5-mL microcentrifuge tubes for 15-min ice incubation. Protein homogenate preparation was then achieved by centrifugation at high speed and low temperature (12,000 rpm, 4 °C, 30 min). Protein quantification proceeded via BCA assay (Thermo Fisher Scientific). Subsequently, supernatants underwent 95 °C thermal denaturation (10 min) prior to SDS-PAGE separation and PVDF membrane electrotransfer. Post-blocking with rapid blocking buffer, membranes received primary antibody incubation (4 °C, overnight; see Supplementary Table 3) followed by species-matched HRP-conjugated secondary antibodies. A chemiluminescence enhancer solution was added to the membrane, and the protein bands were exposed on a chemiluminescence instrument. The relative quantitative data of the immunoblotting were analyzed using ImageJ software.

### Calcium quantification

The reagent used for calcium quantification was a commercial kit (Leagene, China). After PBS washing, VSMCs were incubated overnight after being subjected to 0.6 mol/L HCl. The calcium concentration was measured by collecting the supernatant from the hydrochloric acid solution. Aortic homogenates underwent centrifugation to collect supernatants for calcium quantification. Data normalization revealed proportional calcium-to-protein ratios across samples.

### Alizarin red staining

After PBS washing, VSMCs underwent 15-min fixation in 4% paraformaldehyde prior to three distilled water rinses. Subsequently, alizarin red solution (Leagene, China) was applied for 30 min. For aortic tissue processing, immersion fixation in 4% formaldehyde proceeded for 24 h, after which dehydration in 95% ethanol (24 h) was performed. Terminal staining with 0.003% alizarin red solution lasted 30 h. The red color after washing indicates that calcification occurred.

### Reverse transcription and quantitative real-time polymerase chain reaction (qPCR)

The TRIzol method, implemented with RNAiso Plus reagent (TaKaRa, 9109), served to extract total RNA in this study. Real-time PCR utilized SYBR Green Premix (TaKaRa) as the detection chemistry. Amplification data were monitored and gene expression profiles were analyzed employing the Roche LightCycler 480 platform. Gene expression levels of the target were normalized to GAPDH and analyzed through the 2ΔΔCt method. Supplementary Table 4 contains the primer sequences utilized in this study.

### Immunohistochemistry (IHC)

Following paraformaldehyde fixation and paraffin embedding, 5 μm sections of mouse aortic and human radial artery tissues were prepared. Mouse IHC specimens originated from the descending aorta. Post-sectioning, tissues underwent xylene dewaxing and graded alcohol rehydration. Subsequent to a 30-mi interval, samples were treated with 3% H₂O₂ under ambient conditions and antigen retrieval was then performed with ethylenediaminetetraacetic acid (EDTA) under microwave irradiation. Blocking was performed (5% BSA, 1 h, RT) prior to 16–18 h primary antibody incubation at 4 °C targeting relevant epitopes (Table S3). Subsequent secondary antibody staining (1 h, ambient temperature) preceded diaminobenzidine-based imaging. Image acquisition utilized a Nikon NiU optical microscope, while protein expression quantification relied on ImageJ software.

### Statistical analysis

Statistical analyses were performed using GraphPad Prism 9.0 or SPSS 26.0. Continuous variables are presented as mean ± standard deviation or median (IQR), whereas categorical data appear as frequency counts (percentages). Student’s t-test (normal distributions) or Mann-Whitney U test (non-parametric) was applied. To analyze multiple groups, either one-way ANOVA with Bonferroni correction or the Kruskal-Wallis test, was applied. Bivariate correlations were quantified via the Spearman coefficient, and influencing factors of VC were identified through backward conditional logistic regression modeling.

## Supplementary information

Supplementary Materials

Supplementary Materials

Supplementary_data of the complete signedstamped protocol with methodological documentation of English Version

## Data Availability

RNA-seq data are publicly accessible via NCBI GEO (Accession: GSE279148). (https://www.ncbi.nlm.nih.gov/geo/). The manuscript and supplementary files include all the supporting datasets.
