# Supplementary material for: Inhibition of long interspersed nuclear element-1 by nucleoside reverse transcriptase inhibitors attenuates vascular calcification
Source: Signal Transduct Target Ther. 2025 Oct 1;10:321. doi: 10.1038/s41392-025-02396-4 (PMC12484660; doi:10.1038/s41392-025-02396-4)
Supplement: Supplementary file 1 — Supplementary Materials [file 41392_2025_2396_MOESM1_ESM.pdf]

Supplementary Materials for

Inhibition of long interspersed nuclear element-1 by nucleoside reverse transcriptase inhibitors attenuates vascular calcification

Jianshuai Ma1, Dayu He1, Mingxuan Zhang1, Ziting Zhou1, Jinkun Cheng1, Aoran Huang1, Yaxin Lian1, Yuncong Shi1, Changming Xie1, Zhengyan Guan1, Zhengzhipeng Zhang1, Chen Xie1, Tingting Zhang1*, and Hui Huang1*

Correspondence to: Hui Huang, Email: huangh8@mail.sysu.edu.cn.; Tingting Zhang, Email: zhangtt73@mail.sysu.edu.cn.

This PDF file includes:

Materials and Methods
Figure legends
Figures. S1 to S7
Tables S1 to S4

Materials and Methods

Von kossa staining:

In order to determine the occurrence of VC, the dewaxed and rehydrated vessel sections were incubated with 5% silver nitrate solution and exposed to ultraviolet light until the end of color development. The next step is to treat the section with 5% sodium thiosulfate. The brown spots in the smooth muscle cell layer represent the occurrence of VC, and the range and number represent the severity of calcification. Image collection is carried out using an optical microscope (NikonNiU).

Renal function quantification in mice:

The detection of renal function in mice included renal fibrosis test and serum creatinine test. For the determination of renal fibrosis, the kidney was fixed with paraformaldehyde and embedded in paraffin. The sagittal sections of the kidney were used for morphological staining and fibrosis staining. Morphological staining using hematoxylin and eosin staining kit (Solarbio, China), fibrosis staining using Masson trichromatic staining kit (Solarbio, China), staining according to the manufacturer's instructions. Image collection was carried out using an optical microscope (NikonNiU). Creatinine levels were measured using a modified Jaffé method, in which creatinine reacts with picric acid under alkaline conditions to form a red-colored complex. The absorbance of this complex, which is directly proportional to the creatinine concentration, was then quantified at a wavelength of 495 nm.

Nuclear-cytoplasmic separation:

Nuclear and cytoplasmic separation was carried out according to the previously reported method22. To put it simply, the cells were collected in a 1.5ml centrifuge tube and centrifuged to get cell precipitation. The cells were re-suspended in cytoplasmic lysis buffer and rested on the ice for 20 minutes. The supernatant was taken as cytoplasmic part after low temperature and highspeed centrifugation (4 ℃, 15000g for 5min). The remaining cell precipitates were suspended in the nuclear cleavage buffer and 60min was cleaved on ice. The supernatant was taken as nuclear component after low temperature and highspeed centrifugation (4 ℃, 15000g for 15min).

DNA measured

After nuclear-cytoplasmic separation, the components were treated with Proteinase K and RNase A, followed by phenol-chloroform extraction to remove proteins and RNA. DNA was then precipitated with ethanol. After dilution with distilled water, the DNA was subjected to qPCR analysis. The primers used for qPCR are listed in Supplementary Table S2.

Enzyme-linked immunosorbent assay:

The levels of cGAMP, IFN-a, IFN- β, IL-1, IL-6 and NF- κ B were detected by linked immunosorbent assay (Invitrogen, USA). Enzyme linked immunosorbent assay was carried out in accordance with the manufacturer's instructions and quantified by microplate readers.

RNA-sequencing and data analysis

VSMCs exposed to Pi and transfected with si-scr or si-LINE1, or treated with NRTIs or not. The RNA-sequencing experiments were performed by GENEWIZ, Inc (Hangzhou, China). Briefly,

total RNA was extracted from hVSMCs by Trizol reagent (TaKaRa, Japan, 9109). NEBNext Ultra RNA Library Prep Kit for Illumina (NEB, Ipswich, MA, USA) were used to prepared mRNA libraries following the manufacturer's instructions. RNA sequencing was then performed on the Illumina NovaSeq platform (Illumina, San Diego, CA, USA), and 150 bp paired-end reads were generated. Quality-filtered reads were aligned to the human reference genome using HISAT2 (v2.0.5).7 Read counts for each gene were obtained with featureCounts (v1.5.0-p3) and were standardized as fragments per kilobase million (FPKM). Differentially expressed genes (DEGs) were identified with DESeq2 (v1.20.0) using a false discovery rate (FDR) < 0.05. Heatmaps were generated to visualize gene expression patterns. Kyoto Encyclopedia of Genes and Genomes (KEGG) pathway analysis of the DEGs was implemented. The KEGG pathways with FDR<0.05 were considered significantly enriched. The raw data of RNA-sequencing have been deposited in the NCBI Gene Expression Omnibus.

Figure legends

Figure. S1.

LINE1 is positively related to VC in patients with CKD. (A-D) Correlation of LINE1 mRNA level with age (n = 37), VC scores (n = 25), serum ALP (n = 37), serum Ca^{2+}(n = 37) and serum Pi^{+}(n = 37) levels in CKD patients (Pearson correlation coefficient R value and P value). (E) Immunofluorescence staining quantification for LINE1 in calcified and noncalcified radial artery sections from CKD patients (n=6 per group). Statistical significance was assessed using 2-tailed t tests (e), and Pearson's correlation coefficient analysis (a-d). All values are displayed as means ± SEM.

Figure. S2.

Successful construction of calcified models in VSMC induced by Pi, and CKD and $VitD_3$ overloaded mice. (a-b) Alizarin red staining and calcium content determination showed that Pi induced calcified VSMCs (n =6 per group). (c-d) Alizarin red staining of full-length aorta and calcium content of arterial tissue from CKD mice (n =3 per group). (e-f) Serum creatinine and Masson staining analysis in CKD mice kidneys (n =4 per group); scale = 100μm. (g-h) Alizarin red staining of full-length aorta and calcium content of arterial tissue from $VitD_3$ overloaded mice (n =3 per group). (i and j) Western blot analysis and quantification of Runx2, OPN, Smoothelin, SM22α and LINE1-ORF1 expressions in calcified mice arteries and control groups (i), (n = 3), or in VSMC induced by Pi for the indicated time (j), (n = 3). Statistical significance was assessed using 2-tailed t tests (d,e and h), 1-way ANOVA followed by Dunnett's test (b and j). All values are displayed as means ± SEM.

Figure. S3.

The efficiency of interference with LINE1 by siRNA and NRTIs. (a-c) Western blot analysis and q-PCR analysis of LINE1 expression in VSMCs transfected with control, mock, si- nc, si-LINE1-1, si-LINE1-2， si- LINE1-3 (n = 3 per group). (d-e) q-PCR analysis of LINE1DNA (n =8 per group) and LINE1 mRNA (n =6 per group) level in VSMCs cultured with Piand NRTI. (f-h) VSMCs incubated with Pi and entecavir (1,5,10,50μM). Alizarin red staining (f), calcium content (g), and western blot analysis (h) expression of Runx2, OPN, Smoothelin, SM22ɑ, and LINE1-ORF1. Statistical significance was assessed using 2-tailed t tests (d and e), 1-way ANOVA followed by Dunnett' s test (b, c and g). All values are displayed as means ± SEM.

Figure. S4.

Effects of NRTI on renal function in CKD mice. (a) BUN assay in serum of CKD mice treated with NRTI (n=4 per group). (b) Masson staining in kidney section of CKD mice treated with NRTI (n=3 per group); scale=100 μm. (c-d) Western blot and quantitative analysis of Fibronectin

and Collagen I in the kidney of CKD mice (n=3 per group). Statistical significance was assessed using 1-way ANOVA followed by Dunnett's test (a and d). All values are displayed as means ± SEM.

Figure. S5.

RNA sequence data for VSMC DEGs enriched in control vs LINE1 inhibition. (a-b) The volcano plot shows DEGs in VSMCs between the two groups respectively. (c-d) Heatmap analysis of contraction factor genes from bulk RNA-seq of VSMCs with si-LINE1, or with NRTI treatment (n = 3 per group). (e-f) KEGG pathway annotation of DEGs in si-LINE1 and NRTI treatment groups resulting from bulk RNA-seq (n =3 per group). One-way ANOVA and two-tailed t-tests were used to determine statistical significance. All values are displayed as means ± SEM.

Figure. S6.

cGAS, STING and inflammation factors were elevated in radial artery sections and the serum of mice with CKD-induced VC. (a) Immunofluorescence staining for cGAS and STING in calcified and noncalcified radial artery sections from CKD patients (n=3 per group); scale =50 μm. (b-f) Enzyme-linked immunosorbent assay of IFN-a, IFN- β, IL-1β, IL-6 and NF-κ B in CKD mice serum (n =4 per group). Statistical significance was assessed using 2-tailed t tests (b-f). All values are displayed as means ± SEM.

Figure. S7.

NRTI alleviates cGAS-STING pathway activation. (a and b) Western blot and quantitative of TBK1, IRF3, p-TBK1 and p-IRF3 in VSMCs incubated with Pi and treated with NRTI (n =3 per group). (c and d) Western blot analysis and quantitative of TBK1, IRF3, p-TBK1 and p-IRF3 in VSMCs incubated with Pi after pre-transfected with si-LINE1 (n =3 per group). (e and f) Western blot analysis and quantitative of TBK1, IRF3, p-TBK1 and p-IRF3 in VSMCs incubated with Pi and treated with NRTI after pre-transfected with Ad-LINE1 (n =3 per group). (g-h) western blot analysis and quantitative expression of cGAS, STING, IRF3, p-TBK1 and p-IRF3 from CKD mice (n = 3per group). (i-j) western blot analysis and quantitative expression of TBK1, IRF3, p-TBK1 and p-IRF3 from CKD mice (n =3per group). (k-o) ELISA of IFN- α, IFN- β, IL-1β, IL-6 and NF- κ B in serum of CKD mice treated with NRTI (n =4 per group). Statistical significance was assessed using 1-way ANOVA followed by Dunnett's test (b, d, f, h, j and k-o). All values are displayed as means ± SEM.

Figures. S1 to S7

Figure. S1.

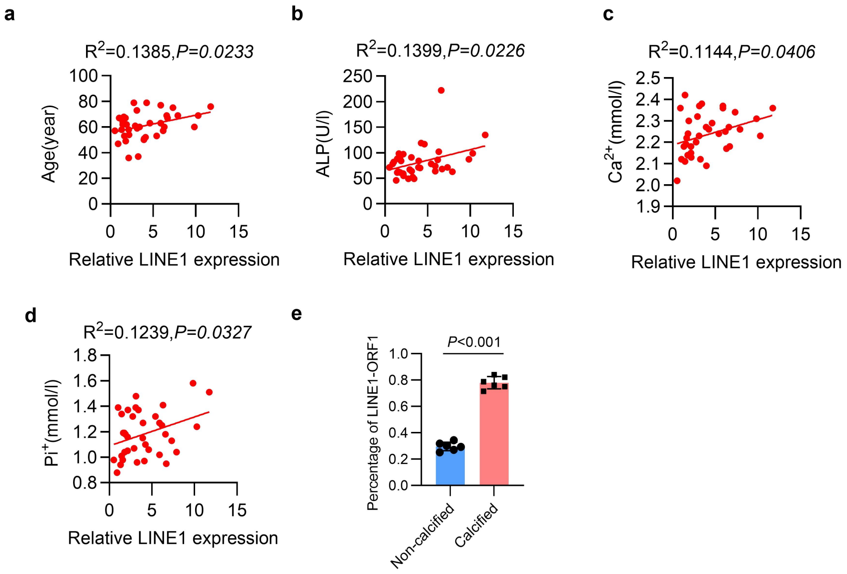

Figure. S2.

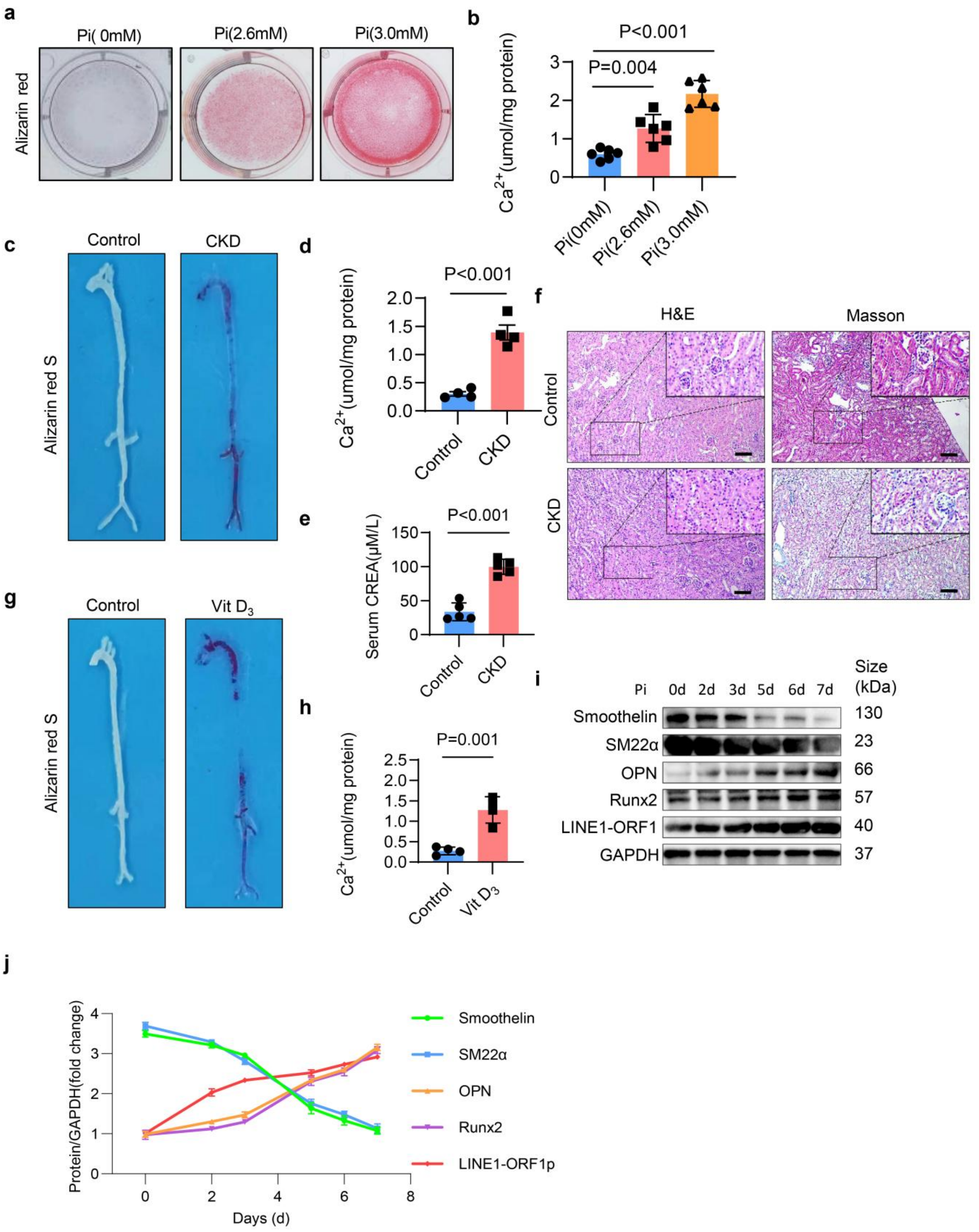
a
Pi(0mM)
Pi(2.6mM)
Pi(3.0mM)
Alizarin red
b
P<0.001
P=0.004
Ca2+(umol/mg protein)
Pi(0mM)
Pi(2.6mM)
Pi(3.0mM)
c
Control
CKD
Alizarin red S
d
P<0.001
Ca2+(umol/mg protein)
Control
CKD
e
P<0.001
Serum CREA(μM/L)
Control
CKD
f
H&E
Masson
Control
CKD
g
Control
Vit D3
Alizarin red S
h
P=0.001
Ca2+(umol/mg protein)
Control
Vit D3
i
Pi
0d 2d 3d 5d 6d 7d
Size (kDa)
Smoothelin 130
SM22α 23
OPN 66
Runx2 57
LINE1-ORF1 40
GAPDH 37
j
Protein/GAPDH(fold change)
Days (d)
Smoothelin
SM22α
OPN
Runx2
LINE1-ORF1p

Figure. S3.

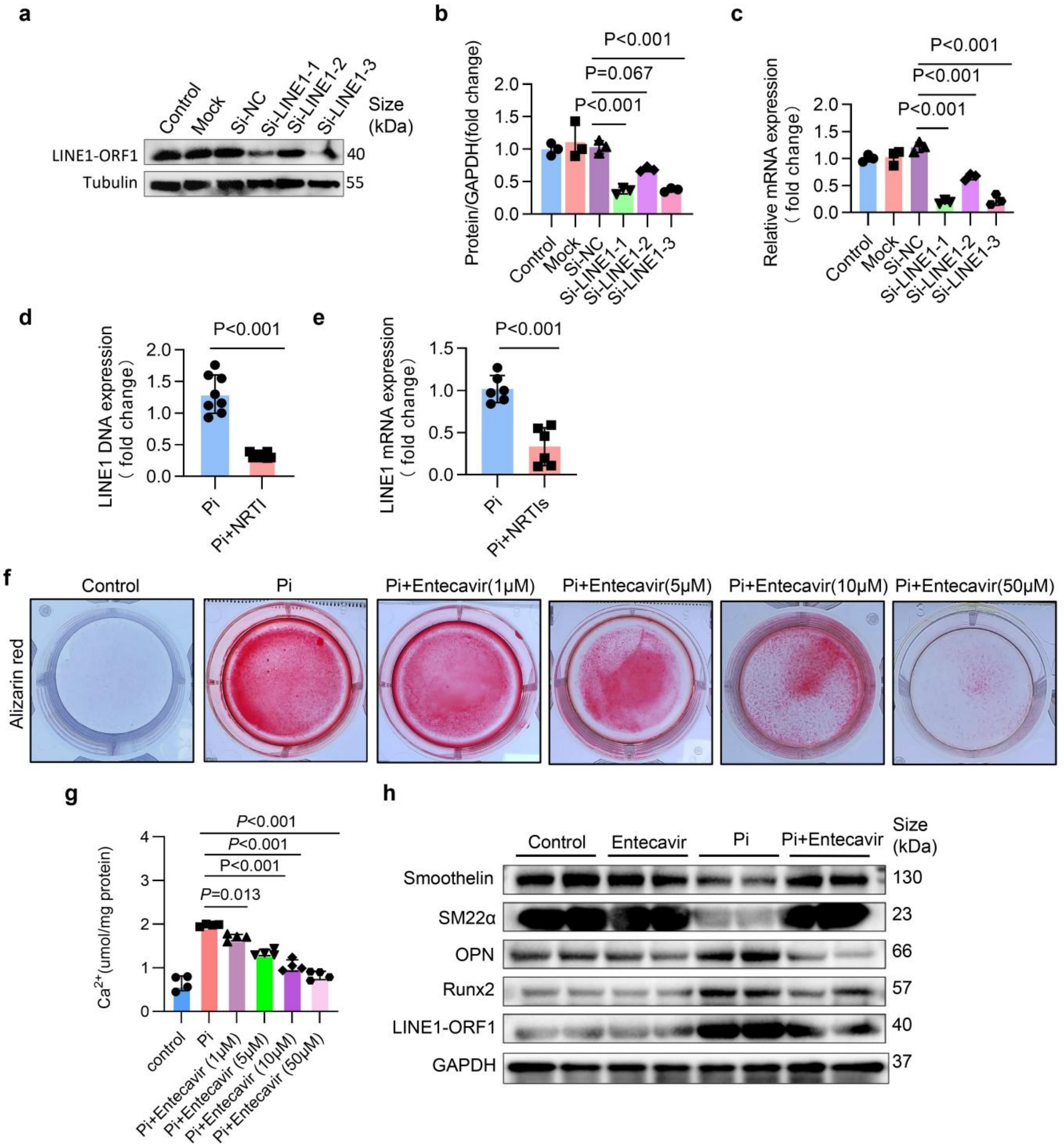
a
Control
Mock
Si-NC
Si-LINE1-1
Si-LINE1-2
Si-LINE1-3
Size (kDa)
LINE1-ORF1
40
Tubulin
55
b
Protein/GAPDH(fold change)
P<0.001
P=0.067
P<0.001
c
Relative mRNA expression (fold change)
P<0.001
P<0.001
P<0.001
d
LINE1 DNA expression (fold change)
P<0.001
Pi
Pi+NRTI
e
LINE1 mRNA expression (fold change)
P<0.001
Pi
Pi+NRTIs
f
Control
Pi
Pi+Entecavir(1μM)
Pi+Entecavir(5μM)
Pi+Entecavir(10μM)
Pi+Entecavir(50μM)
Alizarin red
g
Ca2+(umol/mg protein)
P<0.001
P<0.001
P<0.001
P=0.013
control
Pi
Pi+Entecavir (1μM)
Pi+Entecavir (5μM)
Pi+Entecavir (10μM)
Pi+Entecavir (50μM)
h
Control
Entecavir
Pi
Pi+Entecavir
Size (kDa)
Smoothelin
130
SM22α
23
OPN
66
Runx2
57
LINE1-ORF1
40
GAPDH
37

Figure. S4.

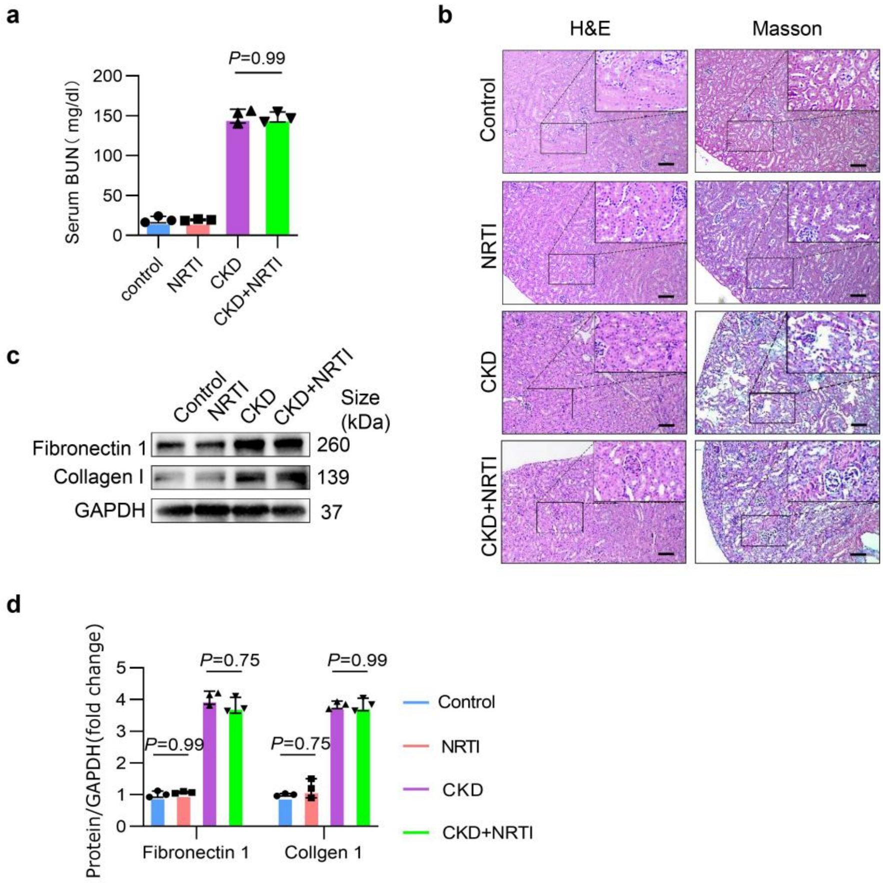
a
P=0.99
Serum BUN (mg/dl)
200
150
100
50
0
control
NRTI
CKD
CKD+NRTI
b
H&E
Masson
Control
NRTI
CKD
CKD+NRTI
c
Control
NRTI
CKD
CKD+NRTI
Size (kDa)
Fibronectin 1
260
Collagen I
139
GAPDH
37
d
Protein/GAPDH(fold change)
5
4
3
2
1
0
P=0.75
P=0.99
P=0.99
P=0.75
Fibronectin 1
Collgen 1
Control
NRTI
CKD
CKD+NRTI

Figure. S5.

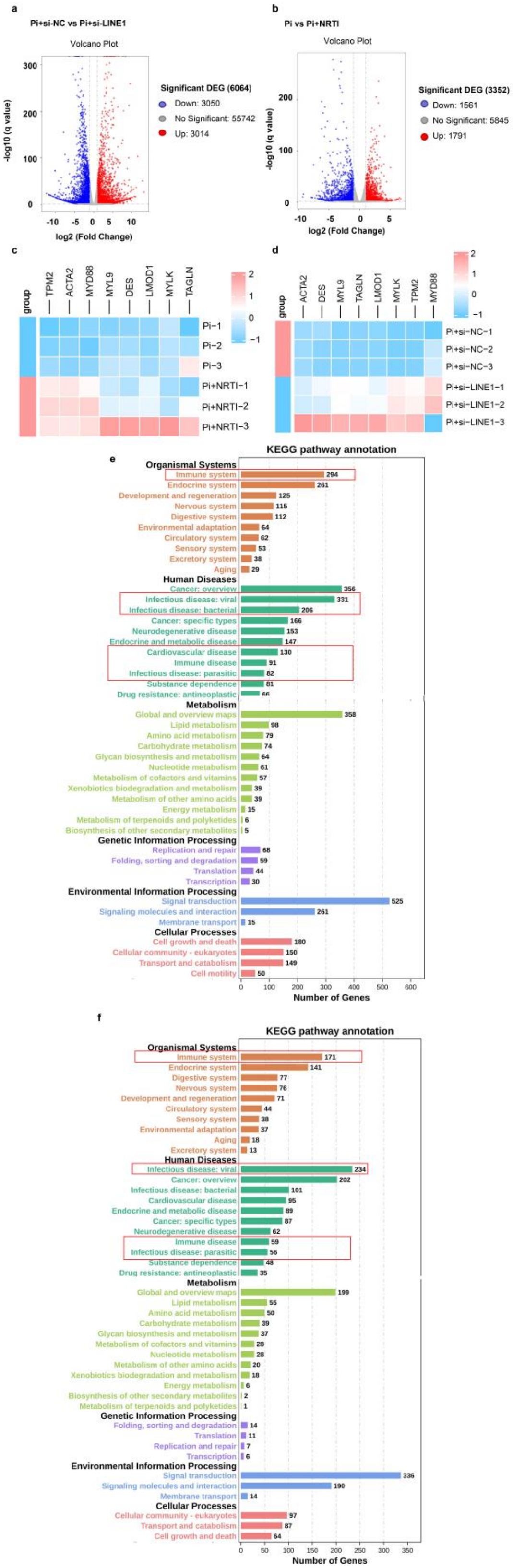
a
Pi+si-NC vs Pi+si-LINE1
Volcano Plot
Significant DEG (6064)
Down: 3050
No Significant: 55742
Up: 3014
-log10 (q value)
log2 (Fold Change)
b
Pi vs Pi+NRTI
Volcano Plot
Significant DEG (3352)
Down: 1561
No Significant: 5845
Up: 1791
-log10 (q value)
log2 (Fold Change)
c
group
TPM2
ACTA2
MYD88
MYL9
DES
LMOD1
MYLK
TAGLN
Pi−1
Pi−2
Pi−3
Pi+NRTI−1
Pi+NRTI−2
Pi+NRTI−3
d
group
ACTA2
DES
MYL9
TAGLN
LMOD1
MYLK
TPM2
MYD88
Pi+si−NC−1
Pi+si−NC−2
Pi+si−NC−3
Pi+si−LINE1−1
Pi+si−LINE1−2
Pi+si−LINE1−3
e
KEGG pathway annotation
Organismal Systems
Immune system 294
Endocrine system 261
Development and regeneration 125
Nervous system 115
Digestive system 112
Environmental adaptation 64
Circulatory system 62
Sensory system 53
Excretory system 38
Aging 29
Human Diseases
Cancer: overview 356
Infectious disease: viral 331
Infectious disease: bacterial 206
Cancer: specific types 166
Neurodegenerative disease 153
Endocrine and metabolic disease 147
Cardiovascular disease 130
Immune disease 91
Infectious disease: parasitic 82
Substance dependence 81
Drug resistance: antineoplastic
Metabolism
Global and overview maps 358
Lipid metabolism 98
Amino acid metabolism 79
Carbohydrate metabolism 74
Glycan biosynthesis and metabolism 64
Nucleotide metabolism 61
Metabolism of cofactors and vitamins 57
Xenobiotics biodegradation and metabolism 39
Metabolism of other amino acids 39
Energy metabolism 15
Metabolism of terpenoids and polyketides 6
Biosynthesis of other secondary metabolites 5
Genetic Information Processing
Replication and repair 68
Folding, sorting and degradation 59
Translation 44
Transcription 30
Environmental Information Processing
Signal transduction 525
Signaling molecules and interaction 261
Membrane transport 15
Cellular Processes
Cell growth and death 180
Cellular community - eukaryotes 150
Transport and catabolism 149
Cell motility 50
Number of Genes
f
KEGG pathway annotation
Organismal Systems
Immune system 171
Endocrine system 141
Digestive system 77
Nervous system 76
Development and regeneration 71
Circulatory system 44
Sensory system 38
Environmental adaptation 37
Aging 18
Excretory system 13
Human Diseases
Infectious disease: viral 234
Cancer: overview 202
Infectious disease: bacterial 101
Cardiovascular disease 95
Endocrine and metabolic disease 89
Cancer: specific types 87
Neurodegenerative disease 62
Immune disease 59
Infectious disease: parasitic 56
Substance dependence 48
Drug resistance: antineoplastic 35
Metabolism
Global and overview maps 199
Lipid metabolism 55
Amino acid metabolism 50
Carbohydrate metabolism 39
Glycan biosynthesis and metabolism 37
Metabolism of cofactors and vitamins 28
Nucleotide metabolism 28
Metabolism of other amino acids 20
Xenobiotics biodegradation and metabolism 18
Energy metabolism 6
Biosynthesis of other secondary metabolites 2
Metabolism of terpenoids and polyketides 1
Genetic Information Processing
Folding, sorting and degradation 14
Translation 11
Replication and repair 7
Transcription 6
Environmental Information Processing
Signal transduction 336
Signaling molecules and interaction 190
Membrane transport 14
Cellular Processes
Cellular community - eukaryotes 97
Transport and catabolism 87
Cell growth and death 64
Number of Genes

Figure. S6.

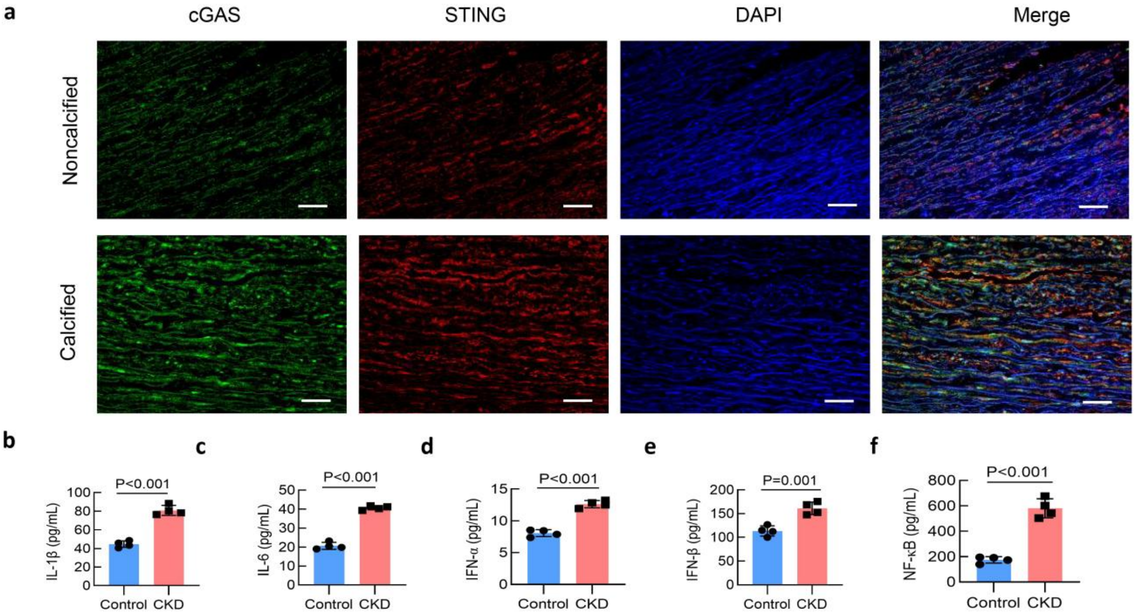
a
cGAS
STING
DAPI
Merge
Noncalcified
Calcified
b
IL-1β (pg/mL)
P<0.001
Control CKD
c
IL-6 (pg/mL)
P<0.001
Control CKD
d
IFN-α (pg/mL)
P<0.001
Control CKD
e
IFN-β (pg/mL)
P=0.001
Control CKD
f
NF-κB (pg/mL)
P<0.001
Control CKD

Figure. S7.

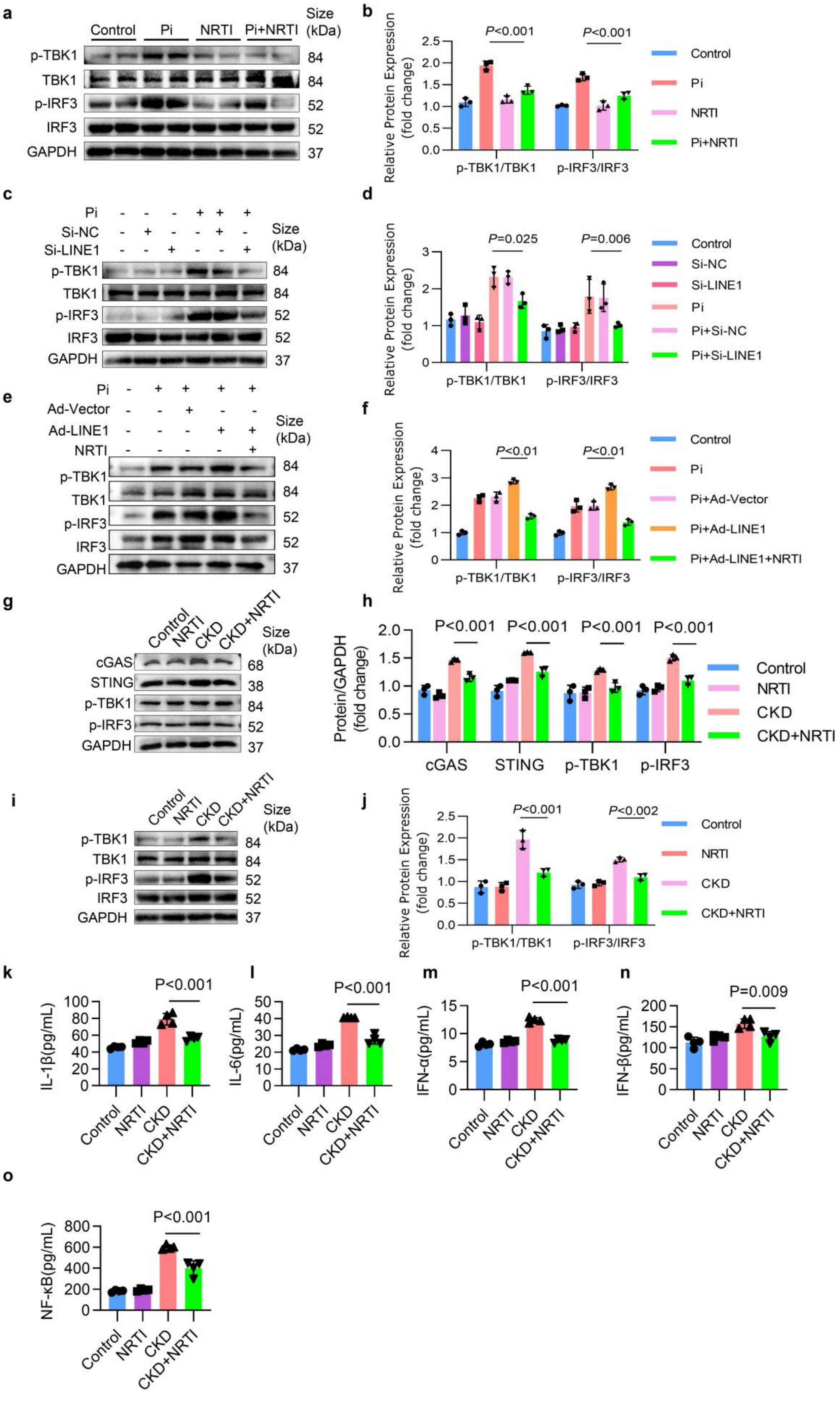
a
Control
Pi
NRTI
Pi+NRTI
Size (kDa)
p-TBK1 84
TBK1 84
p-IRF3 52
IRF3 52
GAPDH 37
b
Relative Protein Expression (fold change)
P<0.001
P<0.001
p-TBK1/TBK1
p-IRF3/IRF3
Control
Pi
NRTI
Pi+NRTI
c
Pi - - - + + +
Si-NC - + - - + -
Si-LINE1 - - + - - +
Size (kDa)
p-TBK1 84
TBK1 84
p-IRF3 52
IRF3 52
GAPDH 37
d
Relative Protein Expression (fold change)
P=0.025
P=0.006
p-TBK1/TBK1
p-IRF3/IRF3
Control
Si-NC
Si-LINE1
Pi
Pi+Si-NC
Pi+Si-LINE1
e
Pi - + + + +
Ad-Vector - - + - -
Ad-LINE1 - - - + +
NRTI - - - - +
Size (kDa)
p-TBK1 84
TBK1 84
p-IRF3 52
IRF3 52
GAPDH 37
f
Relative Protein Expression (fold change)
P<0.01
P<0.01
p-TBK1/TBK1
p-IRF3/IRF3
Control
Pi
Pi+Ad-Vector
Pi+Ad-LINE1
Pi+Ad-LINE1+NRTI
g
Control
NRTI
CKD
CKD+NRTI
Size (kDa)
cGAS 68
STING 38
p-TBK1 84
p-IRF3 52
GAPDH 37
h
Protein/GAPDH (fold change)
P<0.001
P<0.001
P<0.001
P<0.001
cGAS
STING
p-TBK1
p-IRF3
Control
NRTI
CKD
CKD+NRTI
i
Control
NRTI
CKD
CKD+NRTI
Size (kDa)
p-TBK1 84
TBK1 84
p-IRF3 52
IRF3 52
GAPDH 37
j
Relative Protein Expression (fold change)
P<0.001
P<0.002
p-TBK1/TBK1
p-IRF3/IRF3
Control
NRTI
CKD
CKD+NRTI
k
IL-1β(pg/mL)
P<0.001
l
IL-6(pg/mL)
P<0.001
m
IFN-α(pg/mL)
P<0.001
n
IFN-β(pg/mL)
P=0.009
o
NF-κB(pg/mL)
P<0.001
Control
NRTI
CKD
CKD+NRTI

Table S1.
Baseline characteristics of CKD patients with or without VC

Variables	Non-VC (n=12)	VC (n=25)	*P* Value
Age, years	60.25±9.333	61.52±11.07	0.734
Sex(male), n (%)	4（33.3%）	14（56.0%）	0.197
BMI,kg/m2(range)	22.02± 2.10	24.47 ± 3.19	0.021
SBP,mmHg	121.83±17.76	134.29±19.35	0.089
DBP,mmHg	77.50±8.95	76.41±10.60	0.774
eGFR, mL/min/1.37m^2	15.15 (6.18, 25.33)	6.00 (3.55, 28.40)	0.227
CREA,µmol/L	389.15 (178.00,624.05)	675.7 (207.75, 1114.95)	0.160
UA,µmol/L	377.17 ±130.86	399.16±99.74	0.574
Ca, mmol/L	2.22±0.11	2.25±0.09	0.383
Pi, mmol/L	1.13±0.16	1.20±0.19	0.262
ALP, U/L	80.00（61.50，88.00）	71.00（63.50，94.00）	0.936
CHOL,mmol/L	4.79 ± 1.14	4.80 ± 1.42	0.971
TG,mmol/L	1.31（1.09，1.59）	1.22（0.84，2.94）	0.962
LDL-C,mmol/L	2.97 ± 0.85	2.97 ± 1.01	0.993
HDL-C,mmol/L	1.23 (1.02, 1.36)	1.03 (0.90, 1.34)	0.203
WBC (X10^9/L)	6.37 (5.40, 8.79)	6.92 (5.82, 8.09)	0.987

HGB(g/L)	130.00 ± 10.33	125.68 ± 18.44	0.456
ALT(U/L)	15.00 (11.25,25.00)	17.00 (13.00, 25.00)	0.554
AST(U/L)	19.00 (16.25, 29.75)	20.00 (18.00, 27.00)	0.643
hs-CRP (mg/L)	1.97 (0.36,3.10)	1.66 (0.55,4.62)	0.446
LINE1	1.61（1.13，1.88）	4.63（3.12，6.64）	<0.001
VC Agatston score	0（0，0）	231.79（138.67，719.66）	<0.001

Continuous values are expressed as mean ± SD or median (25th to 75th quartiles) for and categorical variables are *n* (%), respectively. Student's *t*-test (all characteristics except sex) or nonparametric Mann-Whitney *U* test (sex) were used to assess statistical significance. BMI, body mass index; SBP, systolic blood pressure; DBP, diastolic blood pressure; eGFR, estimated glomerular filtration rate; UA, Uric Acid; ALP, alkaline phosphatase; CR, creatinine; HDL, high-density lipoprotein; LDL, low-density lipoprotein; hs-CRP, high sensitivity-C reactive protein.

Table S2.
The Relative si-RNA in this work

Name	species	sequences
siRNA-LINE1-1	Homo	TCAGCAATGGAAGATGAAATGAATG
siRNA-LINE1-2	Homo	GAAATGAAGCGAGAAGGGAAG
siRNA-LINE1-3	Homo	AAGAAATGAGCAAAGCCTCCAAGAA
siRNA-cGAS-1	Homo	GGAGCUACUAUGAGCACGUTT
siRNA-cGAS-2	Homo	CCCUGCUGUAACACUUCUUTT
siRNA-cGAS-3	Homo	GGCUAUCCUUCUCUCAUT
siRNA-NC	Homo	GAAGAAGGAGCGGAAGAAGTTATTA

Table S3.
The antibodies used in this study

The antibodies used for western blotting			
Target	Source	Identifiers	Dilution Rate
Anti-ORF1p Rabbit mAb	Cell Signaling Technology	88701	1:1,000
Anti-LINE-1 ORF1p	Abcam	ab216324	1:1,000
Anti-RUNX2	Abcam	ab236639	1:1,000
Anti-OCN	Proteintech	16157-1-AP	1:1,000
Anti-Smoothelin	Abcam	ab219652	1:2,000
Anti-SM22α	Abcam	ab213273	1:1,000
Anti-cGAS	Abcam	ab302617	1:1,000
cGAS Polyclonal antibody	Proteintech	29958-1-AP	1:1,000
Anti-STING	Abcam	ab239074	1:1000
TMEM173/STING Polyclonal antibody	Proteintech	19851-1-AP	1:1000
Phospho-TBK1/NAK-S172 Rabbit pAb	abclonal	AP0847	1:1,000
Phospho-IRF3-S396 Rabbit mAb	abclonal	AP1412	1:1000
Anti-GAPDH	Proteintech	60004-1-Ig	1:5,000
Anti-Tublin	Proteintech	11224-1-AP	1:1,000
**The antibodies used for Immunohistochemistry staining**			
Target	Source	Identifiers	Dilution Rat
Anti-ORF1p Rabbit mAb	Cell Signaling Technology	88701	1:200
Anti-LINE-1 ORF1p	Abcam	ab216324	1:200
Anti-RUNX2	Abcam	ab236639	1:200
**The antibodies used for Immunofluorescent staining**			
Anti-LINE-1 ORF1p	Abcam	ab245249	1:100

Anti-RUNX2	Abcam	ab192256	1:500
Anti-cGAS	Abcam	ab302617	1:50
Anti-STING	Abcam	ab239074	1:100

Table S4.
Primers for q-PCR

**Name**	**sequences**
GAPDH Forward	AGCCACATCGCTCAGACAC
GAPDH Reverse	GCCCAATACGACCAAATCC
LINE1 Forward	AGGAAAGCCCATCAGACTAACAGT
LINE1 Reverse	GGCCTGGTGGTGACAAAATCT
IL1β Forward	GCCAGTGAAATGATGGCTTATT
IL1β Reverse	AGGAGCACTTCATCTGTTTAGG
IL6 Forward	CACTGGTCTTTTGGAGTTTGAG
IL6 Reverse	GGACTTTTGTACTCATCTGCAC
NF-κB Forward	ATGTCCGCGTCCCACTAGCA
NF-κB Reverse	GCCCCACGCCCTGTTTCTTT
IFNA Forward	GCCTCGCCCTTTGCTTTACT
IFNA Reverse	CTGTGGGTCTCAGGGAGATCA
IFNE Forward	GGCCTCTACCACTATCTTCTCTC
IFNE Reverse	ACACTGCTGAATTGACAAGGTTT
