# Supplementary material for: Inhibition of long interspersed nuclear element-1 by nucleoside reverse transcriptase inhibitors attenuates vascular calcification
Source: Signal Transduct Target Ther. 2025 Oct 1;10:321. doi: 10.1038/s41392-025-02396-4 (PMC12484660; doi:10.1038/s41392-025-02396-4)

Supplementary Materials for

Inhibition of long interspersed nuclear element-1 by nucleoside reverse transcriptase inhibitors attenuates vascular calcification

Jianshuai Ma1, Dayu He1, Mingxuan Zhang1, Ziting Zhou1, Jinkun Cheng1, Aoran Huang1, Yaxin Lian1, Yuncong Shi1, Changming Xie1, Zhengyan Guan1, Zhengzhipeng Zhang1, Chen Xie1, Tingting Zhang1*, and Hui Huang1*

Correspondence to: Hui Huang, Email: huangh8@mail.sysu.edu.cn.; Tingting Zhang, Email: zhangtt73@mail.sysu.edu.cn.

This PDF file includes:

Unedited blot and gel images

Unedited blot and gel images

Figure 2c

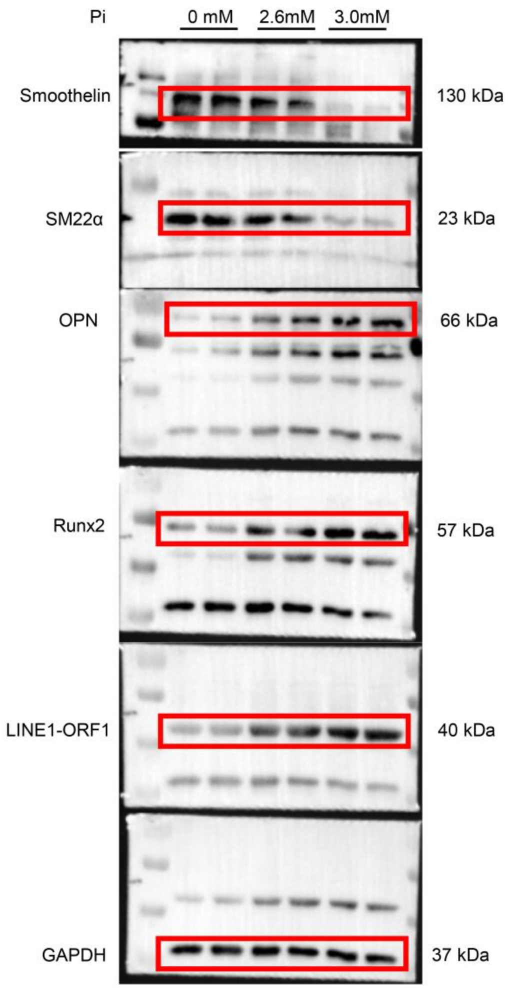

Figure 2e

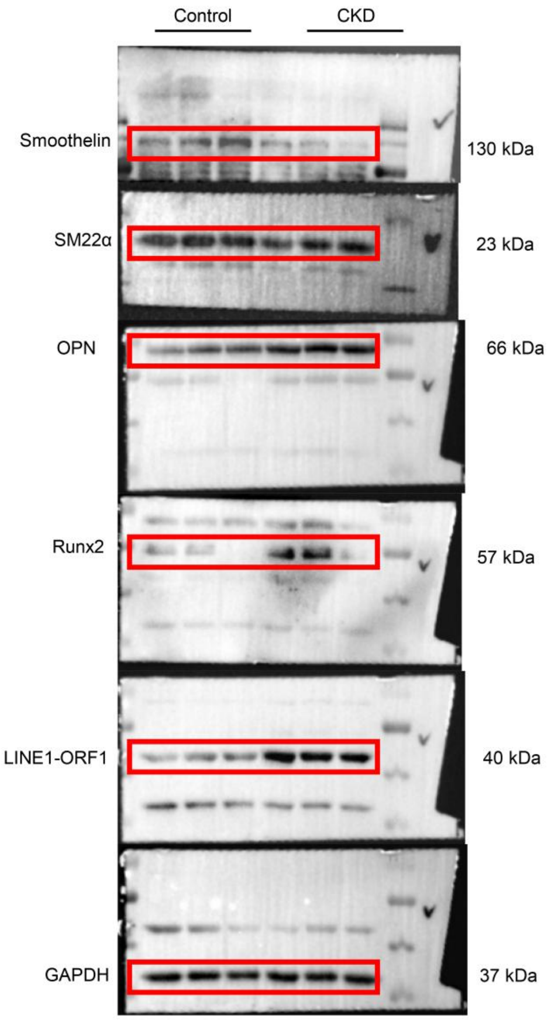

Control
CKD
Smoothelin
130 kDa
SM22α
23 kDa
OPN
66 kDa
Runx2
57 kDa
LINE1-ORF1
40 kDa
GAPDH
37 kDa

Figure 2h

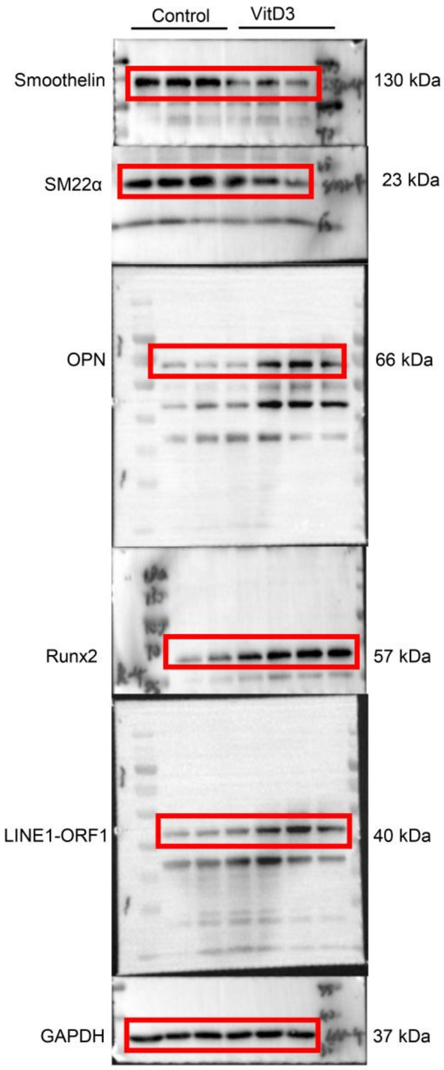

Control
VitD3
Smoothelin
130 kDa
SM22α
23 kDa
OPN
66 kDa
Runx2
57 kDa
LINE1-ORF1
40 kDa
GAPDH
37 kDa

Figure 3c

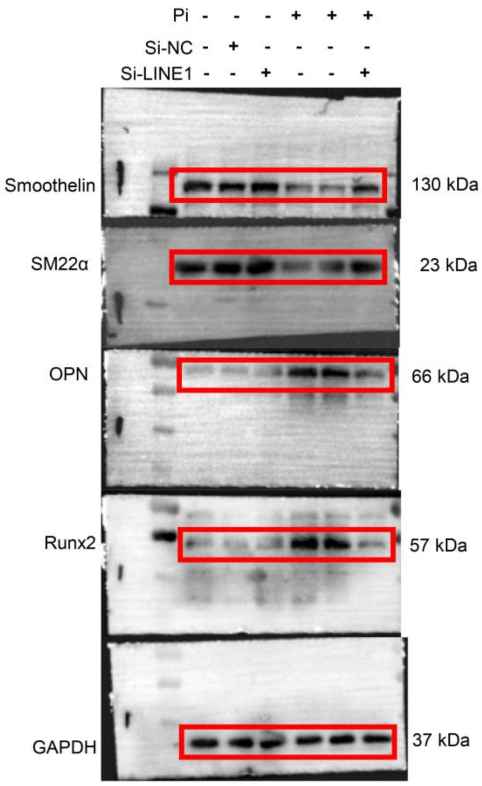

Pi - - - + + +
Si-NC - + - - - -
Si-LINE1 - - + - - +
Smoothelin
130 kDa
SM22α
23 kDa
OPN
66 kDa
Runx2
57 kDa
GAPDH
37 kDa

Figure 3g

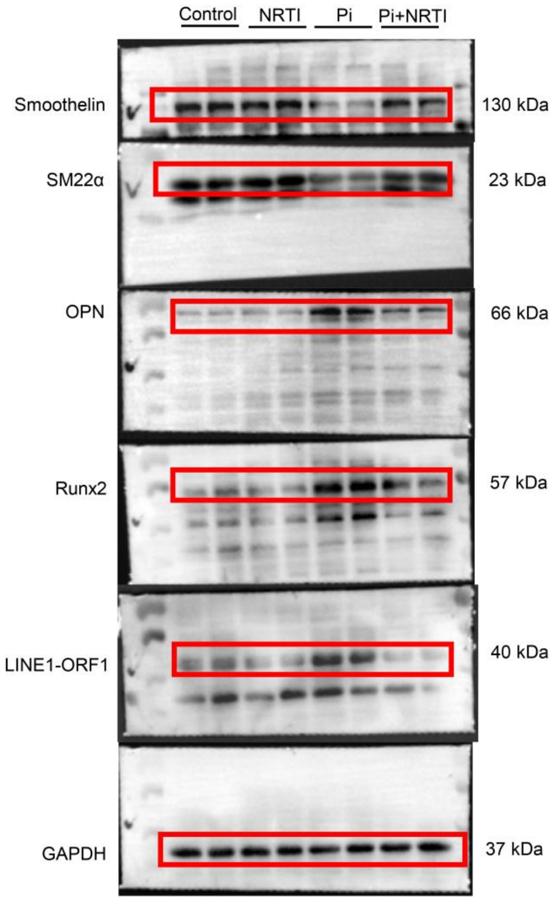

Control
NRTI
Pi
Pi+NRTI
Smoothelin
130 kDa
SM22α
23 kDa
OPN
66 kDa
Runx2
57 kDa
LINE1-ORF1
40 kDa
GAPDH
37 kDa

Figure 4k

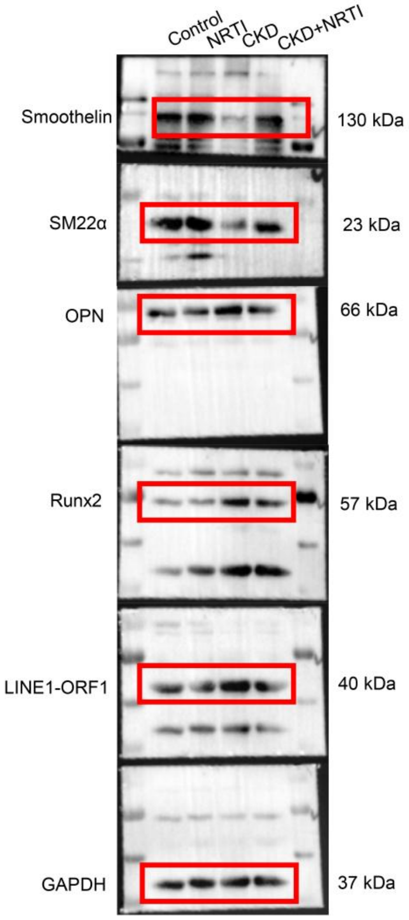

Control
NRTI
CKD
CKD+NRTI
Smoothelin
130 kDa
SM22α
23 kDa
OPN
66 kDa
Runx2
57 kDa
LINE1-ORF1
40 kDa
GAPDH
37 kDa

Figure 4m

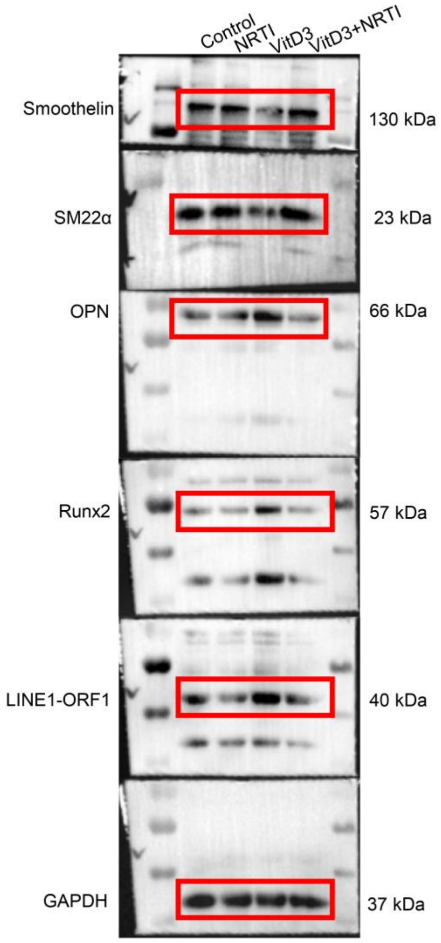

Control
NRTI
VitD3
VitD3+NRTI
Smoothelin
130 kDa
SM22α
23 kDa
OPN
66 kDa
Runx2
57 kDa
LINE1-ORF1
40 kDa
GAPDH
37 kDa

Figure 6a

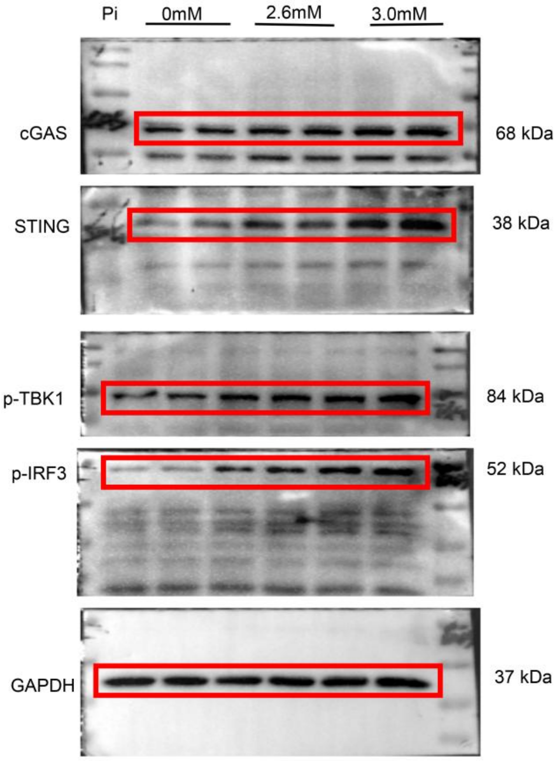

Pi
0mM
2.6mM
3.0mM
cGAS
68 kDa
STING
38 kDa
p-TBK1
84 kDa
p-IRF3
52 kDa
GAPDH
37 kDa

Figure 6j

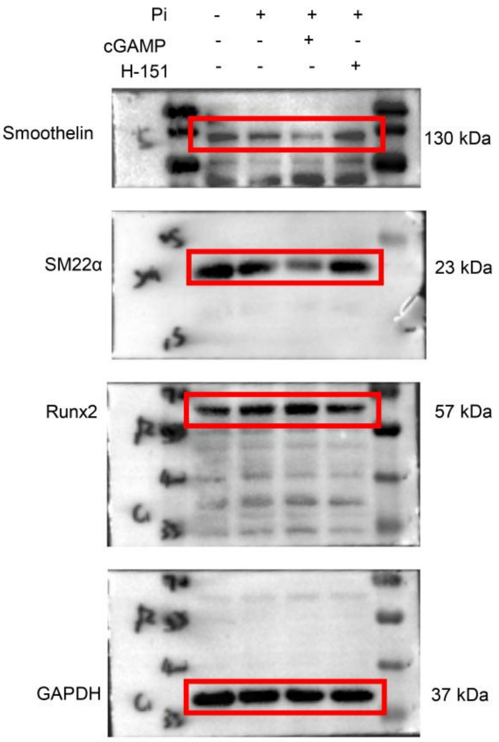

Pi - + + +
cGAMP - - + -
H-151 - - - +
Smoothelin
130 kDa
SM22α
23 kDa
Runx2
57 kDa
GAPDH
37 kDa

Figure 7a

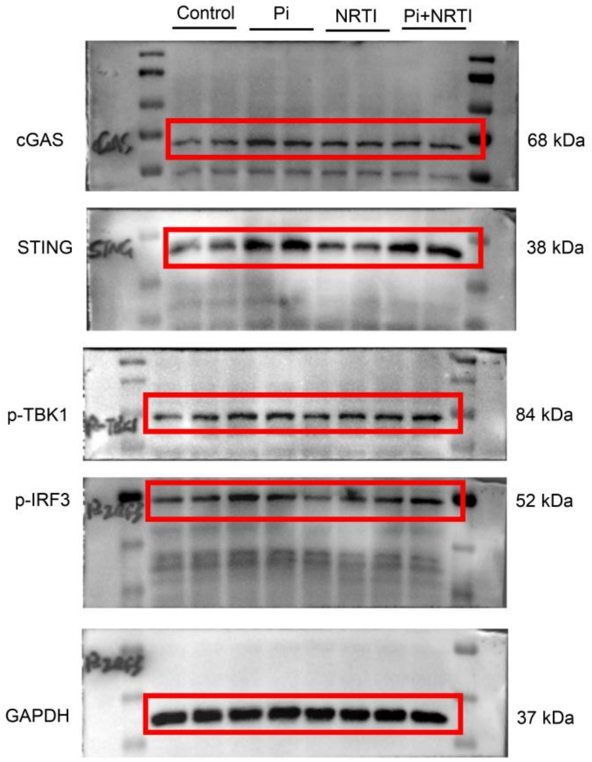

Control
Pi
NRTI
Pi+NRTI
cGAS
68 kDa
STING
38 kDa
p-TBK1
84 kDa
p-IRF3
52 kDa
GAPDH
37 kDa

Figure 7c

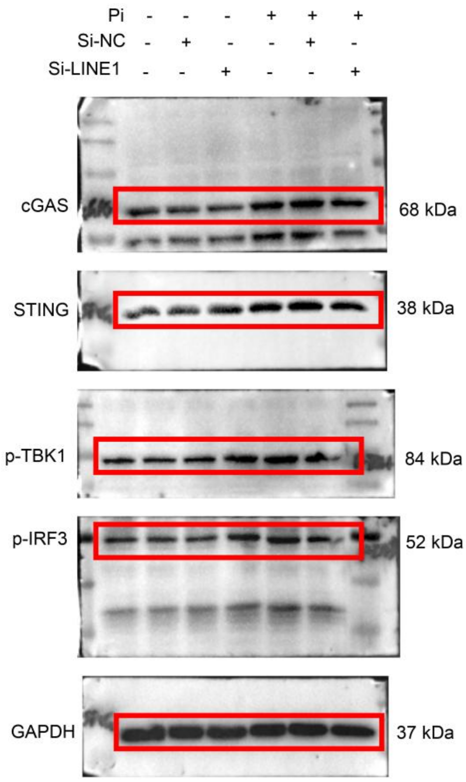

Pi - - - + + +
Si-NC - + - - + -
Si-LINE1 - - + - - +
cGAS
68 kDa
STING
38 kDa
p-TBK1
84 kDa
p-IRF3
52 kDa
GAPDH
37 kDa

Figure 7e

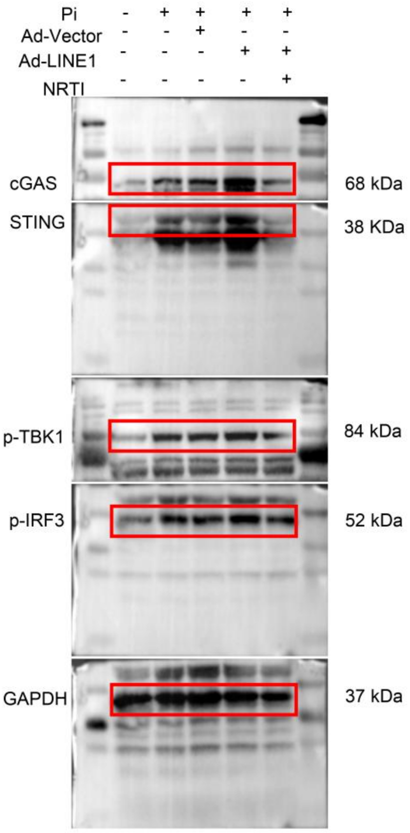
Pi - + + + +
Ad-Vector - - + - -
Ad-LINE1 - - - + +
NRTI - - - - +
cGAS
68 kDa
STING
38 KDa
p-TBK1
84 kDa
p-IRF3
52 kDa
GAPDH
37 kDa

Figure 7m

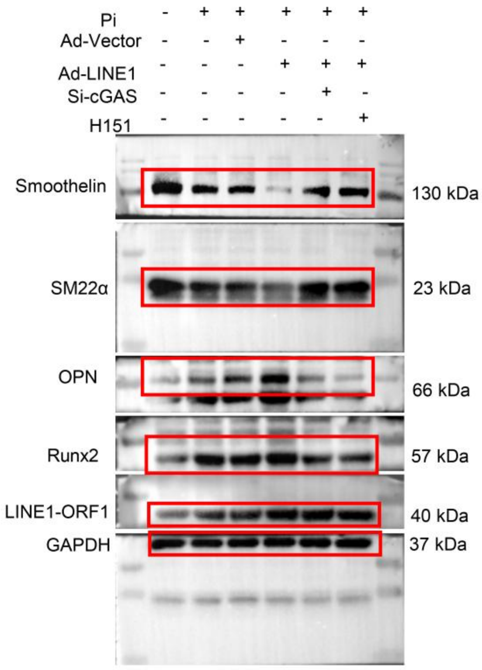

Pi - + + + + +
Ad-Vector - - + - - -
Ad-LINE1 - - - + + +
Si-cGAS - - - - + -
H151 - - - - - +
Smoothelin
130 kDa
SM22α
23 kDa
OPN
66 kDa
Runx2
57 kDa
LINE1-ORF1
40 kDa
GAPDH
37 kDa

Supplementary figure 2i

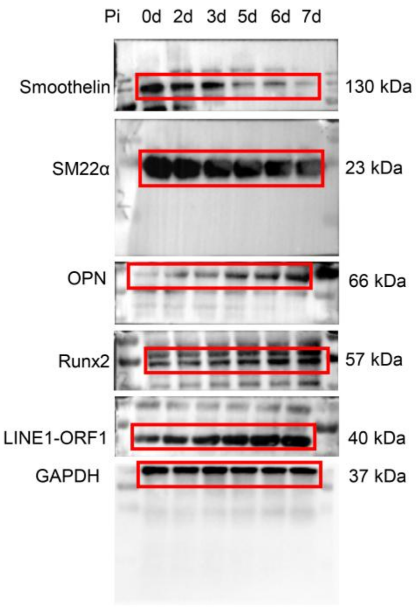

Supplementary figure 3a

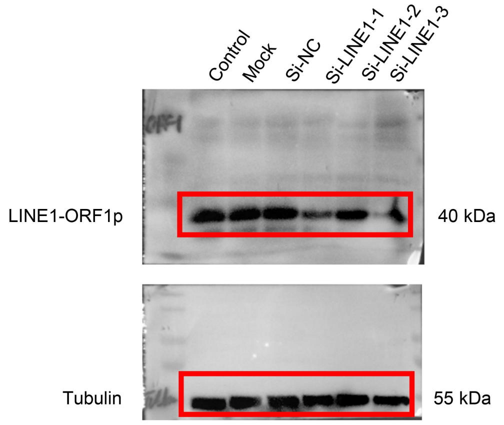

Supplementary figure 3h

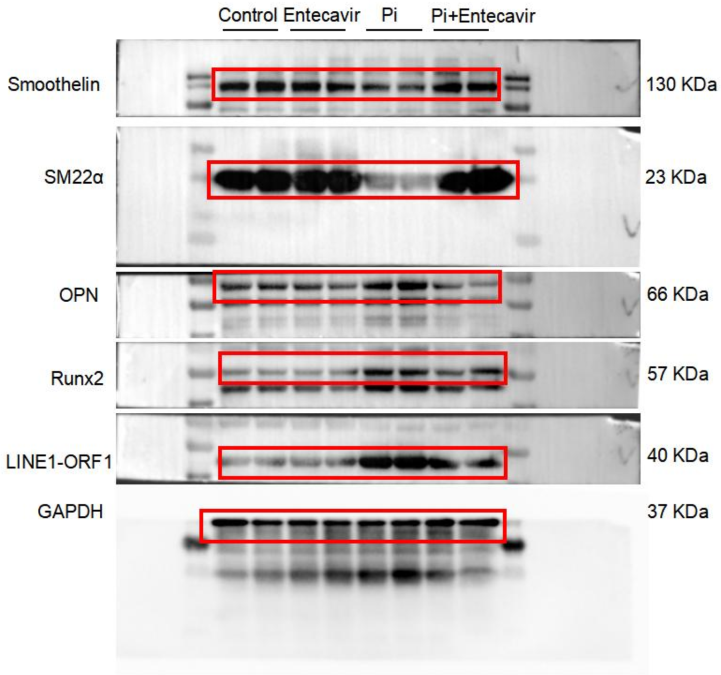

Supplementary figure 4c

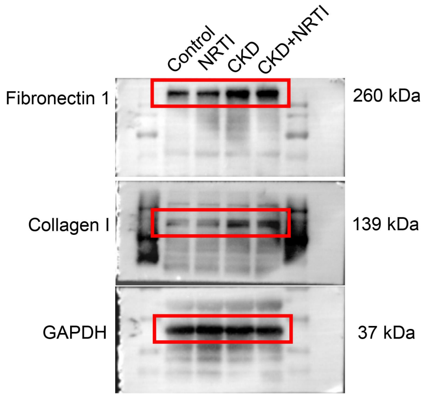

Supplementary figure 7a

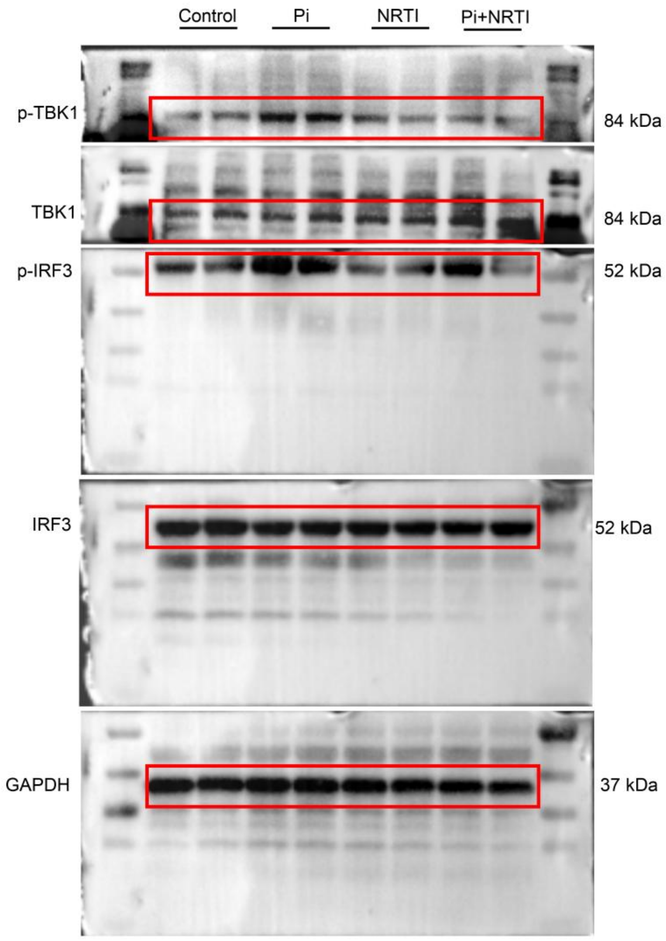

Supplementary figure 7c

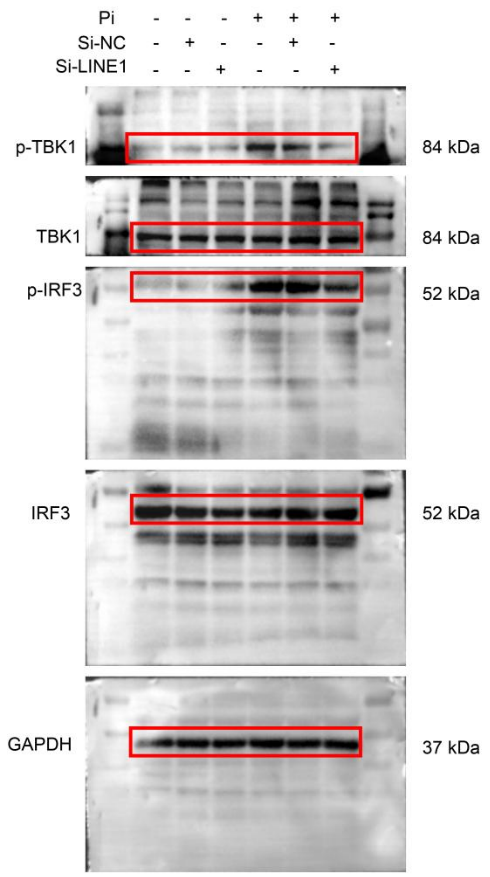

Supplementary figure 7e

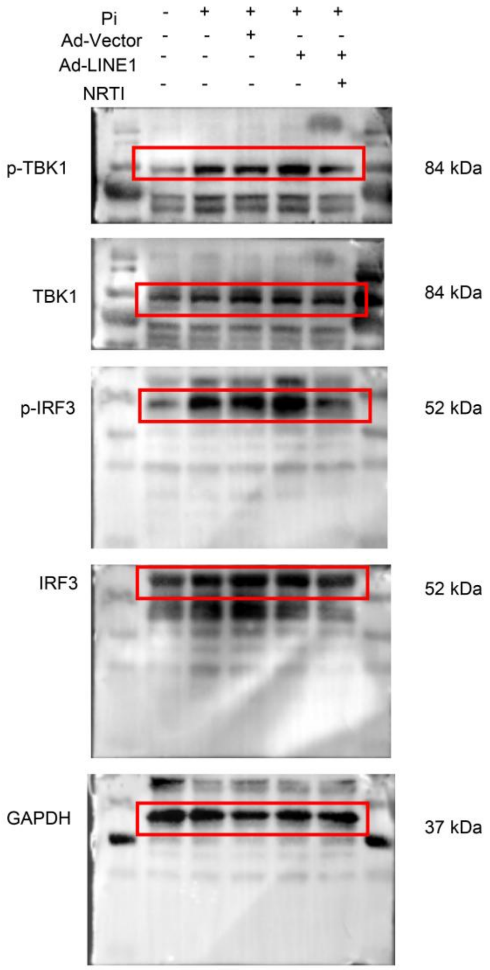

Supplementary figure 7g

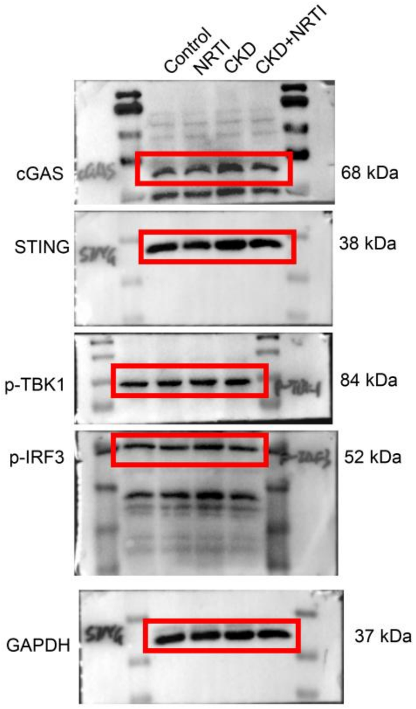

Supplementary figure 7i

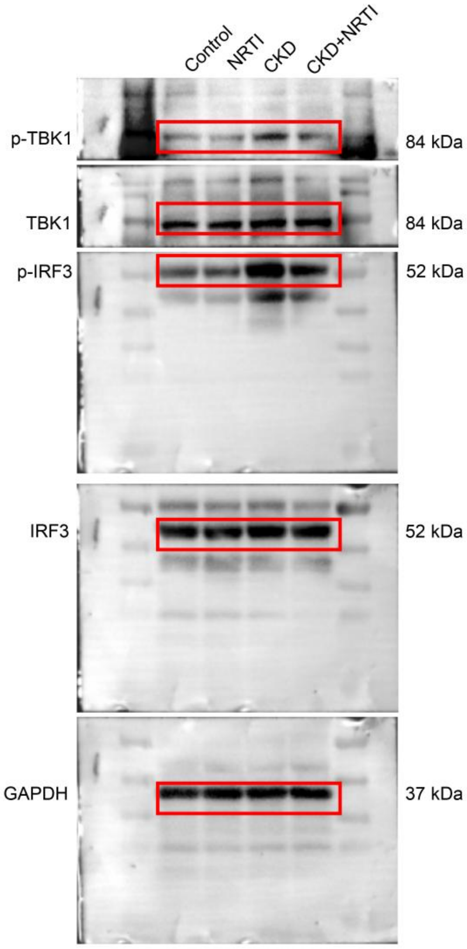

Supplement: Supplementary file 2 — Supplementary Materials [file 41392_2025_2396_MOESM2_ESM.pdf]
