# Supplementary material for: Inhibition of long interspersed nuclear element-1 by nucleoside reverse transcriptase inhibitors attenuates vascular calcification
Source: Signal Transduct Target Ther. 2025 Oct 1;10:321. doi: 10.1038/s41392-025-02396-4 (PMC12484660; doi:10.1038/s41392-025-02396-4)
Supplement: Supplementary file 3 — Supplementary_data of the complete signedstamped protocol with methodological documentation of English Version [file 41392_2025_2396_MOESM3_ESM.pdf]

The Eighth Affiliated Hospital of Sun Yat-sen University (Futian, Shenzhen)

Clinical Research Project Application Form Initiated by the Investigator

Institution Acceptance Number: ZDBY-IIT-202409-170   Date of Filling: 11 Sep, 2024

<table>
<tr><td>Project Name</td><td colspan="5">Study on the efficacy of nucleotide reverse transcriptase inhibitors in the treatment of vascular calcification</td></tr>
<tr><td>Sponsor</td><td>The Eighth Affiliated Hospital of Sun Yat-sen University (Futian, Shenzhen)</td><td>The Responsible Person of the Sponsor</td><td colspan="3">Hui Huang</td></tr>
<tr><td>In-hospital PI</td><td>Hui Huang</td><td>Responsible Department</td><td>Cardiovascular Medicine Department</td><td>Contact Information</td><td>13923730765</td></tr>
<tr><td>Project Contact Person</td><td>Jianshuai Ma</td><td>Contact Information (Telephone and E-mail)</td><td colspan="3">892986433@qq.com</td></tr>
<tr><td>Research Grants</td><td colspan="5">□Enterprises □ Government □ Foundations □ International Organizations
■ Graduate Projects □ Our Institute □ Others( )
Name of Funder: Shenzhen Science and Technology Innovation Commission---[Shenzhen Key Laboratory of Precision Prevention and Control of Major Chronic Diseases and Metabolic Research] (only project funds will be used)
Funding amount: 50,000 yuan
□No funding, please provide a statement of no funding</td></tr>
<tr><td>Study Duration</td><td colspan="5">1 year (from the date of initial ethics approval)</td></tr>
<tr><td rowspan="3">Study Type</td><td rowspan="2">□ Interventional Study</td><td colspan="4">□ Randomized controlled study
□ Non-randomized controlled study</td></tr>
<tr><td colspan="4">□ Use within the Labeling
□ Off-label drug use
□ Overdose
□ Add indications
□ Expand the routes of drug administration
□ Off-label use □ Over-represented population
□ Others( )</td></tr>
<tr><td>■ Observational Study</td><td colspan="4">□Case report/case series report
□Cross-sectional study □Case-control study □Cohort study
■Retrospective case studies
□Diagnostic studies □Others ( )</td></tr>
</table>

<table>
<tr><td></td><td colspan="5">□High-risk study □ Medium-risk study ■ Low-risk study
Note: High-risk study: interventional research with off-label drugs, new surgical procedures, traumatic examinations, or biological treatments, etc., targeting special populations such as minors (<18 years old), elderly patients (>65 years old) and pregnant women; Medium-risk study: studies between high and low risk; Low-risk study: general observational studies</td></tr>
<tr><td>Research Scope</td><td colspan="5">□International □ Domestic ■ In the hospital</td></tr>
<tr><td>Our Institute's Participation Form</td><td colspan="5">■Independent □ As team leader
□Participate. Team leader's unit and person in charge: ____________</td></tr>
<tr><td rowspan="2">Participating Organization</td><td colspan="3"></td><td>PI</td><td></td></tr>
<tr><td colspan="3"></td><td>PI</td><td></td></tr>
<tr><td colspan="6">Participants (Including PIs)</td></tr>
<tr><td>Name</td><td>Academic Degree</td><td>Institution/Department</td><td>Job Title/Position</td><td>GCP or Ethics Training Certificate (Yes or No).</td><td>Project Division</td></tr>
<tr><td>Hui Huang</td><td>Doctor</td><td>Cardiovascular Medicine Department</td><td>Professor</td><td>Yes</td><td>Project Leader</td></tr>
<tr><td>Summary of the scenario (Significance of the purpose of the study, design type, case and control settings, randomization method, intervention measures, observation indicators, observation endpoints, and follow-up).</td><td colspan="5">Objective: To explore the efficacy of nucleoside reverse transcriptase inhibitor therapy on vascular calcification. Design type: Retrospective case study: Research subjects: Patients diagnosed with AIDS or hepatitis B from October 2013 to October 2023 in the Department of Infectious Diseases, Eighth Affiliated Hospital of Sun Yat-sen University (Futian, Shenzhen) [2000 cases are planned to be included];
Observation indicators: According to the inpatient order system, the patient's medication history and treatment status are collected. The basic information of patients collected from the inpatient case system includes, including age, gender, diabetes, CKD, CVD, total cholesterol, triglycerides, low-density lipoprotein, high-density lipoprotein, apolipoprotein, creatinine, total bilirubin, direct bilirubin, aspartate aminotransferase, alanine aminotransferase, hemoglobin, platelets and chest CT or coronary CT imaging, etc.;
A retrospective case study was conducted to explore the relationship between nucleoside reverse transcriptase inhibitors and vascular calcification, and to analyze the effect of nucleoside reverse transcriptase inhibitors on vascular calcification by univariate and multivariate logistic regression, and to explore the efficacy of nucleoside reverse transcriptase inhibitor treatment on vascular calcification. The research results of this project will validate a new therapeutic strategy of nucleoside reverse transcriptase to inhibit vascular inflammation and reduce the socio-economic and health burden caused by vascular calcification.</td></tr>
</table>

Applicant Statement

This project is conducted under the premise of not harming the rights and interests of the subjects. It strictly follows the "Regulations on the Management of Human Genetic Resources of the People's Republic of China", "Good Clinical Practice (GCP) for Drug Clinical Trials", the "Non-registered Clinical Research Operation Management System and Procedures of Our Hospital", and other relevant laws and regulations. The provided materials are all true and reliable, and the research process is guaranteed to be standardized.

This project does not involve administrative approval of the collection, preservation, utilization, and external provision of human genetic resources. Projects that meet the relevant management regulations of human genetic resources in our country must be approved by the science and technology administrative department of the State Council before conducting research.

This project has not received any funding. The intellectual property rights (including the release of results, paper writing, paper publication, enjoyment of specific research results, etc.) belong to the Eighth Affiliated Hospital of Sun Yat-sen University (Futian, Shenzhen), and the Eighth Affiliated Hospital of Sun Yat-sen University (Futian, Shenzhen) shall bear the liability for compensation when the subject suffers research-related damages.

Signature of the project leader:

11 Sep, 2024

Department Opinions	■Agree □ Disagree<br><br>Signature of the department director: Date:
Clinical Research Department Opinion	■Meet the requirements of the project and agree to submit it to the Ethics Committee for review<br>□ Disagree to proceed with the project<br>□After modification, the project will be re-approved for review<br><br>Head of clinical research department (signed/stamped)<br><br>Date:

Remark:1. The application form needs to be printed on both sides. 2. Fill it outDescription: Please fill in ■, error demonstration: ☑、√、●

Clinical Study Protocol

Project name: Study on the Efficacy of Nucleotide Reverse Transcriptase Inhibitors in the Treatment of Vascular Calcification

Project leader: Hui Huang

Sponsor: The Eighth Affiliated Hospital, Sun Yat-sen University

Department: Department of Cardiology

Contact Information: +86 13923730765

Study protocol confirmation signature page

Version number: 3.0

Version date: 31 October 2024

Title: Study on the Efficacy of Nucleotide Reverse Transcriptase Inhibitors in the Treatment of Vascular Calcification

In accordance with the Chinese Good Clinical Practice regulations, I commit to diligently fulfill my responsibilities as the Investigator. I will personally conduct or provide direct supervision for this clinical study. I have thoroughly reviewed this protocol and agree to discharge all related duties in compliance with Chinese law, the *Declaration of Helsinki*, Chinese GCP regulations, and this study protocol. Any modifications to the protocol will be implemented only after approval by the Ethics Committee, except when immediate action is necessary to protect the safety, rights, and interests of the research subjects.

Primary Investigative Site: The Eighth Affiliated Hospital, Sun Yat-sen University

Principal Investigator (Print): Principal Investigator (signature):

Hui Huang

Date: 31 October 2024

1.Protocol Abstract

Study Objectives: To explore the efficacy of nucleoside reverse transcriptase inhibitor therapy on vascular calcification

Study Type: Cross-Sectional Study

Study Population: Patients diagnosed with Hepatitis B or Acquired Immune Deficiency Syndrome (AIDS) in Department of Infectious Diseases, The Eighth Affiliated Hospital, Sun Yat-sen University, October 2013 to October 2023.

Study Endpoints: Data on medication history and treatments were collected from the Hospital Order Entry System. Demographic and clinical data were extracted from the Inpatient Medical Record System, including: age, sex, diabetes mellitus, chronic kidney disease, cardiovascular disease, Total cholesterol, Triglycerides, Low-density lipoprotein cholesterol, High-density lipoprotein cholesterol, Apolipoproteins, Serum creatinine, Total bilirubin, Direct bilirubin, Aspartate aminotransferase, Alanine aminotransferase, Hemoglobin, Platelet count, Chest computed tomography, Coronary computed tomography angiography.

This project will conduct a Cross-Sectional study to investigate the association between nucleoside reverse transcriptase inhibitors (NRTIs) and vascular calcification. Univariate and multivariate logistic regression analyses will assess the effect of NRTIs on vascular calcification and evaluate their therapeutic efficacy. The findings will validate NRTIs as a novel anti-inflammatory strategy for vascular calcification, thereby alleviating its socioeconomic and healthcare burden

2.Study background

Vascular calcification profoundly impacts both quality of life and longevity in affected individuals

Vascular calcification is a pathological process characterized by ectopic deposition of calcium-phosphate as hydroxyapatite crystals within the vascular wall, analogous to physiological bone mineralization [1,2]. This phenomenon

predominantly occurs in aging, diabetes mellitus, and end-stage renal disease [1,2]. While historically regarded as a passive degenerative process, accumulating evidence indicates that vascular calcification resembles skeletal bone formation and represents a highly regulated biological process [3]. The underlying mechanisms involve intricate interactions among multiple factors: oxidative stress and inflammatory responses, apoptosis and matrix vesicle release, vascular injury, hyperphosphatemia, hypercalcemia, deficiency of calcification inhibitors, Osteogenic/chondrogenic inducing factors, and phenotypic switching of vascular smooth muscle cells, these pathways collectively promote mineral deposition within the extracellular matrix, culminating in vascular calcification [4-6].

Vascular calcification induces increased arterial wall stiffness and reduced compliance, thereby contributing to myocardial ischemia, left ventricular hypertrophy, and heart failure. Furthermore, it potentiates thrombus formation and plaque instability, establishing this pathology as a major contributor to the elevated morbidity and mortality observed in cardiovascular and cerebrovascular diseases [7-9]. Ultimately, vascular calcification substantially impairs quality of life and longevity in affected populations. Similarly, a study evaluating the association between coronary artery calcium scoring and cumulative cardiovascular mortality further indicates that vascular calcification serves as a critical risk factor for cardiovascular disease-related deaths [10]. However, current diagnostic and therapeutic strategies targeting vascular calcification remain substantially constrained. Consequently, identifying high-efficacy therapeutics to prevent or retard the initiation and progression of vascular calcification carries significant clinical implications.

Inflammation plays an integral role in the pathogenesis and progression of vascular calcification

Inflammation is recognized as a risk factor for vascular calcification [11]. Elevated circulating inflammatory markers correlate with higher levels of bone

mineral metabolism markers (e.g., FGF-23 and alkaline phosphatase) [12], and associate with increased prevalence, severity, and progression of vascular calcification, suggesting a mechanistic link between inflammation and calcification [13,14]. Experimental studies demonstrate that inflammatory cell activation and cytokine release within the arterial wall trigger oxidative cascades, initiating programmed osteogenic processes — most critically, the osteogenic phenotypic transformation of vascular smooth muscle cells (VSMCs), which ultimately accelerates vascular calcification [15]. Hence, this association reflects a causal relationship wherein inflammation actively contributes to vascular calcification. Confirmation of such causality in humans would yield profound therapeutic implications, fundamentally transforming the clinical paradigm for preventing and treating vascular calcification. Nevertheless, the molecular underpinnings of this linkage remain incompletely elucidated. Intriguingly, emerging evidence indicates that inflammation may serve not only as a stimulus for calcification but also as its consequence, highlighting a self-perpetuating feed-forward loop between inflammatory activation and vascular mineralization [16-18]. Indeed, calcium phosphate crystals can be recognized by macrophages as damage-associated molecular patterns, activating the inflammasome complex — a multiprotein platform that promotes caspase-1-mediated maturation of the pro-inflammatory cytokine interleukin-1β (IL-1β) [19].

Targeting inflammation with NRTIs represents a novel therapeutic strategy to mitigate vascular calcification

NRTIs are clinically approved for treating AIDS and hepatitis B, functioning through blockade of nucleotide incorporation during viral RNA reverse transcription [20-22]. Mounting preclinical evidence demonstrates that NRTIs ameliorate senescence-associated phenotypes by suppressing LINE-1 retrotransposon-driven inflammatory bursts [23-25]. Clinical studies reveal that

NRTI therapy associates with a 33% reduction in diabetes incidence, attributable to its systemic anti-inflammatory effects [26]. Notably, NRTIs have also been shown to attenuate age-related macular degeneration progression via analogous anti-inflammatory mechanisms [27]. Nevertheless, whether NRTIs confer protection against vascular calcification remains unexplored. We therefore investigated the therapeutic potential of NRTIs in vascular calcification.

Therefore, this project will conduct a cross-sectional clinical study to investigate the association between NRTIs use and vascular calcification. Univariate and multivariate logistic regression analyses will be employed to determine the effect of NRTIs on vascular calcification. The study aims to assess the therapeutic efficacy of NRTI treatment against vascular calcification. Successful validation of NRTIs as a novel anti-inflammatory strategy for vascular calcification would alleviate its substantial socioeconomic burden and healthcare costs.

3. Study objectives

To investigate the prevalence and severity of vascular calcification in HIV or hepatitis B patients receiving NRTIs, compared to those not receiving NRTI therapy.

4.Research content and innovation

4.1 This project will conduct a retrospective study to:

Quantify vascular calcification prevalence in patients receiving NRTIs.

Determine whether NRTIs serve as protective factors against vascular calcification and identify specific beneficiary subgroups through stratified analyses.

4.2 Innovation: this represents the first clinical investigation evaluating the therapeutic efficacy of NRTIs against vascular calcification.

5. Study design

5.1 Study site and study population

Study site: Department of Infectious Diseases, The Eighth Affiliated Hospital, Sun Yat-sen University

Study population: Patients diagnosed with Hepatitis B or AIDS, October 2013 to October 2023

Exclusion criteria:

1. Pediatric patients, Pregnant or lactating women.
2. Patients without prior chest CT or coronary CT angiography.
3. Patients with incomplete medication history documentation.

5.2 Sample size calculation

A total of 2,000 subjects will be enrolled, comprising: 1,000 patients receiving NRTIs. 1,000 non-NRTIs patients

Calculation Methodology:

Sample size estimation was performed using the "Mean Difference Between Groups" module in PASS 15 software. The per-group sample size N1 was derived from the formula: $\sigma X = \sigma Diff \times \sqrt{\frac{1}{N} + \frac{1}{N}}$. This initial estimate was then inflated by 10% to account for anticipated loss to follow-up, yielding the final per-group sample size N2.

5.3 Case Report Form (CRF) Data Collection Fields

Data on medication history and treatments were collected from the Hospital Order Entry System. Demographic and clinical data were extracted from the Inpatient Medical Record System, including: age, sex, diabetes mellitus, chronic kidney disease, cardiovascular disease, Total cholesterol, Triglycerides, Low-density lipoprotein cholesterol, High-density lipoprotein cholesterol, Apolipoproteins, Serum creatinine, Total bilirubin, Direct bilirubin, Aspartate aminotransferase, Alanine aminotransferase, Hemoglobin, Platelet count,

Chest computed tomography, Coronary computed tomography angiography.

5.4 Research protocol

Study cohorts were stratified into two groups: Non-NRTIs group: Patients not receiving nucleoside reverse transcriptase inhibitors; NRTIs Group: Patients treated with ≥1 NRTIs (including Abacavir, Emtricitabine, Lamivudine, Didanosine, Zidovudine, Stavudine, Tenofovir). Vascular calcification incidence was compared between groups to evaluate the protective effect of NRTIs against vascular calcification.

5.5 Collection, storage, use, and disposal of research data and/or biological specimens

5.5.1 Data collection

Standardization: utilize standardized data collection tools and forms to ensure consistency and accuracy.

Privacy protection: implement de-identification and anonymization protocols to safeguard participant confidentiality.

5.5.2 Data Storage

Security measures: store data in encrypted databases/systems.

Access control: restrict data access to authorized personnel; conduct quarterly access audits.

Backup procedures: Regular backups shall be performed to prevent data loss or corruption, with backup data protected by equivalent security measures.

5.5.3 Data use

Purpose limitation: Data shall be used exclusively for the research purposes declared at collection, and shall not be utilized for any unauthorized purposes.

Compliance: Adherence to relevant regulations and ethical guidelines, including but not limited to the General Data Protection Regulation and International Council for Harmonisation Good Clinical Practice guidelines.

Reporting and publication: When disseminating research findings, participants'

personal information shall be safeguarded against disclosure in compliance with academic publishing standards and ethical norms.

5.5.4 Data disposal

Plan development: Establish a Data Disposal Plan to ensure appropriate processing when the study concludes or data is no longer required.

Secure destruction: Employ secure destruction methods (e.g., data erasure software, physical destruction of media) to render data irretrievable.

Record retention: Maintain disposal documentation to certify proper data destruction and compliance with applicable requirements.

5.6 Data Management and Statistical Analysis Plan

Data management: Raw data post-detection and imaging materials will be electronically archived.

Statistical analysis: summary statistics will report demographic and baseline characteristics; Categorical variables: expressed as percentages; Continuous variables: summarized using descriptive statistics (mean, median, standard deviation, minimum, maximum). Group comparisons: two groups: Student's t-test or Mann-Whitney U test (non-parametric), multiple groups: One-way ANOVA. Adjusted for confounding variables: univariate and multivariate logistic regression analyses will calculate odds ratios (ORs) with 95% confidence intervals (95% CI) to evaluate the association between NRTIs and vascular calcification.

6 Ethics Review and Informed Consent

6.1 Ethics Committee Review

Protocols and all materials directly related to study participants must be submitted to the Ethics Committee. Formal initiation of the study may commence only after obtaining written approval from the Ethics Committee. Investigators must submit annual progress reports to the Ethics Committee at

least yearly. Upon study termination and/or completion, investigators must provide written notification to the Ethics Committee. All modifications to study procedures (e.g., protocol amendments) must be promptly reported to the Ethics Committee. These changes may not be implemented prior to Ethics Committee approval, except for alterations necessary to eliminate immediate, direct risks to participants. In such cases, the Ethics Committee shall be notified without delay.

6.2 Patient Information and Informed Consent

As a Cross-Sectional study utilizing exclusively clinical care data generated during routine diagnosis and treatment, this project qualifies for waiver of informed consent because: No additional risks are imposed on patients; No adverse impact occurs on participants' rights or health; Confidentiality is legally guaranteed: Patient information will remain protected except when legally mandated for disclosure. Therefore, this study will apply for exemption from obtaining patient informed consent.

7. **Quality Management Plan**

7.1 Data management: Ensure accuracy and completeness of historical data.

7.2 Compliance and documentation: Adhere to regulatory standards and maintain detailed records.

7.3 Risk assessment: Identify and mitigate potential biases and errors in data.

7.4 Data analysis: Implement robust statistical methods and validation techniques.

7.5 Review and auditing: Conduct regular procedure reviews and audits to ensure protocol compliance.

7.6 Training: Provide adequate training for personnel involved in data handling and analysis.

8. **Confidentiality Measures**

Results derived from this study may be published in medical journals. However,

patient confidentiality will be legally protected, and personal information will not be disclosed unless legally mandated. When necessary, regulatory authorities and the Hospital Ethics Committee and their authorized personnel may access patient records in accordance with applicable regulations.

9. **Anticipated Project Timeline and Completion Date**

The research project is anticipated to be completed by 30 June 2025.

10. References

1. Lanzer P, Boehm M, Sorribas V, Thiriet M, Janzen J, Zeller T etal. Medial vascular calcification revisited: review and perspectives. Eur Heart J 2014;35:1515-1525.

2. Giachelli CM. Vascular calcification mechanisms. J Am Soc Nephrol 2004;15:2959-2964.

3. Ouyang L, Su X, Li W, Tang L, Zhang M, Zhu Y etal. ALKBH1-demethylated DNA N6-methyladenine modification triggers vascular calcification via osteogenic reprogramming in chronic kidney disease. JClinInvest2021;131:e146985.

4. Demer LL, Tintut Y. Inflammatory, metabolic, and genetic mechanisms of vascular calcification. Arterioscler Thromb Vasc Biol 2014;34:715-723.

5. Petsophonsakul P, Burgmaier M, Willems B, Heeneman S, Stadler N, Gremse F etal. Nicotine promotes vascular calcification via intracellular Ca2+-mediated, Nox5-induced oxidative stress, and extracellular vesicle release in vascular smooth muscle cells. Cardiovasc Res 2022;118:2196-2210.

6. Singh AP, Sosa MX, Fang J, Shanmukhappa SK, Hubaud A, Fawcett CH et al. α Klotho Regulates Age-Associated Vascular Calcification and Lifespan in Zebrafish. CellRep 2019;28:2767-2776.e5.

7. Gorbunova V, Seluanov A, Mita P, McKerrow W, Fenyö D, Boeke JD etal. The role of retrotransposable elements in ageing and age-associated diseases. Nature 2021;596:43-53.

8. Cordaux R, Batzer MA. The impact of retrotransposons on human genome evolution. Nat Rev Genet 2009;10:691-703.

9. Simon M, Van Meter M, Ablaeva J, Ke Z, Gonzalez RS, Taguchi T et al. LINE1 Derepression in Aged Wild-Type and SIRT6-Deficient Mice Drives Inflammation. CellMetab 2019;29:871-885.e5.

10. De Cecco M, Ito T, Petrashen AP, Elias AE, Skvir NJ, Criscione SW etal. L1 drives IFN in senescent cells and promotes age-associated inflammation. Nature 2019;566:73-78.

11. Li Z, Duan S, Hua X, Xu X, Li Y, Menolfi D etal.Asymmetric distribution of parental H3K9me3 in S phase silences L1 elements. Nature 2023;623:643-651.

12. Brochard T, McIntyre RL, Houtkooper RH, Seluanov A, Gorbunova V, Janssens GE. Repurposing nucleoside reverse transcriptase inhibitors (NRTIs) to slow aging. Ageing Res Rev 2023;92:102132.

13. Shanahan CM. Mechanisms of vascular calcification in CKD-evidence for premature ageing. Nat Rev Nephrol2013;9:661-670.

14. Minton K. Lipid metabolism extinguishes cGAS-STING-induced inflammation. Nat Rev Immunol2023;23:785.

15. Gao P, Ascano M, Wu Y, Barchet W, Gaffney BL, Zillinger T et al. Cyclic [G(2',5')pA(3',5')p] is the metazoan second messenger produced by DNA-activated cyclic GMP-AMP synthase. Cell 2013;153:1094-1107.

16. Shang G, Zhang C, Chen ZJ, Bai XC, Zhang X. Cryo-EM structures of STING reveal its mechanism of activation by cyclic GMP-AMP. Nature 2019;567:389-393.

17. Zhao B, Du F, Xu P, Shu C, Sankaran B, Bell SL etal.A conserved PLPLRT/SD motif of STING mediates the recruitment and activation of TBK1. Nature 2019;569:718-722.

18. Civril F, Deimling T, de Oliveira Mann CC, Ablasser A, Moldt M, Witte G et al. Structural mechanism of cytosolic DNA sensing by cGAS. Nature 2013;498:332-337.

19. Cao DJ, Schiattarella GG, Villalobos E, Jiang N, May HI, Li T etal. Cytosolic DNA Sensing Promotes Macrophage Transformation and Governs Myocardial Ischemic Injury. Circulation 2018;137:2613-2634.

20. Hu D, Cui YX, Wu MY, Li L, Su LN, Lian Z etal.Cytosolic DNA sensor cGAS plays an

essential pathogenetic role in pressure overload-induced heart failure. Am J PhysiolHeartCircPhysiol2020;318 :H1525-H1537.

21. Luo W, Wang Y, Zhang L, Ren P, Zhang C, Li Y etal.Critical Role of Cytosolic DNA and Its Sensing Adaptor STING in Aortic Degeneration, Dissection, and Rupture. Circulation2020;141:42-66.

22. Liang C, Ke Q, Liu Z, Ren J, Zhang W, Hu J et al. BMAL1 moonlighting as a gatekeeper for LINE1 repression and cellular senescence in primates. Nucleic Acids Res 2022;50:3323-3347.

23. Liu A, Chen Z, Li X, Xie C, Chen Y, Su X et al. C5a-C5aR1 induces endoplasmic reticulum stress to accelerate vascular calcification via PERK-eIF2 α -ATF4-CREB3L1 pathway. Cardiovasc Res 2023;119 :2563-2578.

24. Liu Y, Jesus AA, Marrero B, Yang D, Ramsey SE, Sanchez G etal.Activated STING in a vascular and pulmonary syndrome. NEnglJMed2014;371 :507-518.

25. Haag SM, Gulen MF, Reymond L, Gibelin A, Abrami L, Decout A etal. Targeting STING with covalent small-molecule inhibitors. Nature 2018;559:269-273.

26. Gorbunova V, Boeke JD, Helfand SL, Sedivy JM. Human Genomics. Sleeping dogs of the genome. Science2014;346 :1187-1188.

27. Yue Z, Nie L, Ji N, Sun Y, Zhu K, Zou H et al. Hyperglycaemia aggravates periodontal inflamm-aging by promoting SETDB1-mediated LINE-1 de-repression in macrophages. JClinPeriodontol2023;50:1685-1696.
